# The therapeutic effect and specific mechanism involved active Chinese medicine component biochaninA in glioma

**DOI:** 10.3389/fimmu.2026.1780159

**Published:** 2026-04-21

**Authors:** Dan Chai, Mengran Xi, Lu Gao, Lei Sun, Desong Kong, Yujie Zhu, Jinghua Zhang, Qiang Cai, Xinru Xiao, Changqing Wang

**Affiliations:** 1 Nanjing Hospital of Chinese Medicine Affiliated to Nanjing University of Chinese Medicine, Nanjing, Jiangsu, China; 2 Fudan University – Dr. Kong Joint Research Center for Sports Medicine and Health Footwear, Fudan University Institute of Sports Medicine (Jinqiao Laboratory), Shanghai, China; 3 Department of Neurosurgery, The First Affiliated Hospital of Anhui Medical University, Hefei, Anhui, China; 4 Acupuncture and Massage College, Shandong University of Traditional Chinese Medicine, Jinan, Shandong, China; 5 Department of Neurosurgery, Xuanwu Hospital, Capital Medical University, China International Neuroscience Institute (CHINA-INI), National Medical Center for Neurological Disorders, Beijing, China; 6 Institute of Literature in Chinese Medicine, Nanjing University of Chinese Medicine, Nanjing, Jiangsu, China

**Keywords:** biochanin A, glioma, *GSTP1*, immunity, single-cell RNA sequencing, solid tumor, tumor microenvironment

## Abstract

**Introduction:**

Glioma is a common primary solid brain tumor with high incidence and poor prognosis. Biochanin A (bioA), an active component of traditional Chinese medicine, has potential therapeutic effects on it, but its mechanism remains unclear. This study aimed to clarify its mechanism via a multi-omics strategy.

**Methods:**

A multi-omics integration approach was adopted, which combined single-cell RNA sequencing, in vitro experiments, the construction of a Glutathione S-transferase P1 (GSTP1)-based prognostic model, and analyses of immune infiltration and drug sensitivity to evaluate its clinical value.

**Results:**

C2 CENPF⁺ tumor cells were specifically expressed in recurrent gliomas and associated with the bioA pathway. GSTP1 was the key target gene of bioA; its high expression was related to poor prognosis, and its knockout could inhibit glioma progression. The GSTP1-based prognostic model had excellent predictive efficiency.

**Discussion:**

BioA may exert anti-glioma effects by regulating GSTP1, providing theoretical support for its clinical application and a new therapeutic target for glioma.

## Introduction

Neuroglial cells are the root cause of tumor formation. Neurogliomas are the main type of solid tumors in the central nervous system, and their incidence rate is quite high. It is characterized by high heterogeneity, therapeutic resistance, and poor prognosis, and has therefore long been a central focus in neuro-oncology research. According to the revised 2021 World Health Organization (WHO) classification, gliomas are stratified into four distinct grades, ranging from I to IV (1, 2). Among them, the most invasive is grade IV glioblastoma (GBM). The median survival time did not exceed 2 years, and the 5-year survival rate below 5% (3).

The progression of glioma is a complex, multifactorial process closely related to genetic alterations and tumor microenvironment (TME) dysregulation. Previous research has identified several critical molecular events that drive glioma progression, including 10q LOH alteration, EGFR amplification, TP53 mutation, P16INK4a deletion, and PTEN mutation (4–8). In addition, the glioma microenvironment is typified by extensive immune cell infiltration, abnormal vascular architecture, extracellular matrix (ECM) constituents, and a variety of signaling molecules, which act in concert to facilitate malignant cell proliferation and invasion. Although our understanding of glioma pathogenesis has improved, clinical management of this disease still presents considerable difficulties. The marked invasiveness of glioma frequently makes complete surgical resection unachievable. For this reason, concurrent radiotherapy and chemotherapy has become the standard core treatment for glioblastoma (GBM) and other high-grade gliomas, exerting an irreplaceable effect in suppressing local tumor progression and extending patient survival (9) Nonetheless, high-grade gliomas commonly acquire resistance to conventional radiotherapy and chemotherapy (1), and even following standardized treatment (10), tumor recurrence remains inevitable in the majority of patients. In recent years, fungal therapy has emerged as a novel and promising therapeutic strategy for glioma management, and its combined application with standard radiotherapy and chemotherapy has shown potential in reversing treatment resistance and regulating the tumor immune microenvironment (11). From this, it is evident that overcoming immune resistance and developing new and effective treatment methods have become an urgent priority.

The past few years, targeted therapy and immunotherapy have achieved remarkable success in several cancers, including ovarian cancer and melanoma, offering new hope for glioma treatment. With the rapid advancement of precision medicine and multi-omics technologies, single-cell RNA sequencing (scRNA-seq) has enabled the revelation of cellular heterogeneity (12–17). Bulk RNA sequencing (bulk RNA-seq), on the other hand, is instrumental in constructing prognostic models and stratifying patients by risk level, thus facilitating personalized treatment (18–21). Additionally, network pharmacology—a systems biology-based approach with the advantages of multi-target and multi-pathway analysis—has been widely applied to identify potential drug targets and elucidate the pharmacological mechanisms of traditional medicines (22). Integrating scRNA-seq with network pharmacology allows for the identification of novel immune targets within specific malignant subpopulations, thereby accelerating the translation of basic research into clinical applications. Over the past few decades, more and more Chinese medicines have been discovered to have anti-inflammatory and tumor-suppressing capabilities, which may open up new prospects for the treatment of malignant tumors (23–25). A large number of relatively mature active Chinese medicine component have gradually entered preclinical and clinical applications, such as curcumin, artemisinin, and saikosaponin. Experimental studies have demonstrated that curcumin suppresses glioma cell viability through multi-modal mechanisms including G2/M phase cell cycle arrest induction, apoptosis promotion, and autophagy activation (26–28). Artemisinin derivatives exhibit potent anti-glioma effects by inhibiting cellular proliferation and invasive capacity while triggering apoptotic pathways (29, 30). Saikosaponins have also been validated for their anti-glioma pharmacological activity (31). biochanin A (bioA) is a type of isoflavone, possessing multiple biological activities such as antioxidant, anti-inflammatory, antibacterial, and anti-tumor effects, which derived from Chinese medicines like Red Clover Herb (32, 33). Red Clover Herb contains bioactive constituents that exhibit antioxidant and anticancer properties (34). Owing to these multifaceted properties, bioA has emerged as a compelling candidate of interest within contemporary cancer pharmacology (35, 36). Earlier research has confirmed that bioA exerts inhibitory effects on several malignancies, such as breast cancer, osteosarcoma, and lung cancer (37–39). In malignant brain tumors, bioA has also shown certain antitumor potential (40, 41). However, its specific mechanisms of action in glioma remain poorly understood.

It is worth noting that multiple analyses have provided a completely new multi-dimensional framework for the precise treatment of gliomas. By using scRNA-seq to explore the tumor microenvironment and identify key malignant subgroups, the core target genes acting on these subgroups were selected using network pharmacology. A prognostic model was then constructed to analyze immune infiltration and other conditions, thereby providing a solid and systematic foundation for individualized treatment. In summary, the research systematically elucidates the potential mechanisms of bioA in glioma, providing a theoretical basis for precision treatment and novel drug development from Chinese medicine in glioma therapy. The research also provides solid experimental support for Chinese medicine in the prevention and treatment of major and difficult diseases, and clearly explains the scientific connotations involved.

## Methods

### ScRNA-seq data

For the single-cell transcriptomic analysis, we sourced the dataset from the Gene Expression Omnibus (GEO), using the publicly accessible entry GSE182109 as the primary data resource (42). Bulk RNA-seq data were retrieved from the Genomic Data Commons (GDC) data portal associated with The Cancer Genome Atlas (TCGA) (43). All study data were obtained from public databases, so ethical review was not required for this research.

### Quality control of single-cell data

10X Genomics sample data were processed using Seurat (v4.3.0) in R (v4.2.0), with doublets removed using DoubletFinder (v2.0.3). Then we used the following criteria to remove cells that do not meet the standard, as below: 1. Number of detected features (nFeature) < 300 or > 7000; 2. Total number of gene counts (nCount) < 500 or > 100,000; 3. Proportion of mitochondrial gene expression relative to total gene counts > 20% (44).

The NormalizeData function was used to standardize cell data, and the FindVariable Features function could screen the top 2000 hypervariable genes (HVGs). Additionally, the ScaleData function (v3.1.4) was employed to normalize the data, converting the normalized gene expression into Z-scores such that the mean expression of each gene across all cells was 0 and the variance was 1. Batch effect was corrected by Harmony (v0.1.1). The RunPCA function reduced the dimension of the principal component, and any visual display was performed. We applied the FindNeighbors and FindClusters functions to delineate cell populations, while tissue cell identities were determined by evaluating the mean expression patterns of corresponding marker genes. These markers were derived from prior literature and the CellMarker database.

### Cell-cell communication analysis

Glioma intercellular communication (45, 46) was analyzed via “CellChat” (v1.6.1) and CellChatDB.human, focusing on signaling pathways, ligand-receptor pairs, and cofactor-mediated interactions. Furthermore, the “netVisual_diffInteraction” and “Identify Communication Patterns” functions of CellChat were employed to evaluate the situation of cell-cell communications, respectively.

### InferCNV analysis

InferCNV enabled identification of malignant cells. Normal epithelial cells were used as reference cells, and gene expression intensity at different positions in the tumor genome was analyzed to infer chromosomal variations. Glioma cells exhibiting elevated levels of copy number variations (CNVs) were designated as malignant tumor cells (47).

### Pseudotime analysis

Monocle2 (v2.24.0) could explore the pseudotime differentiation trajectories of various cell types in glioma. The CytoTRACE R package (v0.3.3) could predict the differentiation potential of cell types. The Slingshot software (v2.6.0) used minimum spanning trees (MSTs) to infer the lineage differentiation structure and pseudotime changes of glioma cell subgroups. The trajectory curve was drawn using the 'getlineage' and 'getCurves' tools.

### Enrichment analysis

Functional characterization of the identified differentially expressed genes (DEGs) was carried out through enrichment analyses based on the Gene Ontology (GO) and Kyoto Encyclopedia of Genes and Genomes (KEGG) databases. Additionally, Gene Set Enrichment Analysis (GSEA) was utilized to screen for associated biological pathways. Data with an absolute value of normalized enrichment score (NES) > 0.5 and a p-value < 0.05 were considered statistically significant (48–50).

### Cell stemness evaluation

AUCell determined whether subgroups of the input gene sets are enriched in the expressed genes of each cell by calculating the area under the curve (AUC). AUCell was utilized to assess the stemness properties of distinct cell subgroups.

### SCENIC analysis

To infer single-cell regulatory networks, pySCENIC (v0.12.1) was used to reveal the underlying mechanisms. We employed AUCell to quantify the enrichment of transcription factors (TFs) and to evaluate their regulatory activities (51, 52).

### Protein-protein interaction networks

BioA-associated targets were obtained from the traditional Chinese medicine systems pharmacology database and analysis platform (TCMSP) and STRING database (53), and the corresponding PPI network was subsequently constructed. And displayed by Cytoscape 3.9.1 software (54). Then, perform enrichment analysis using the Cytoscape plugin ClueGO (55).

### Risk scoring model

The risk scoring model was constructed using glioma-related datasets obtained from the TCGA database, a core resource of harmonized cancer genomic data. We applied univariate Cox regression to pinpoint genes that showed significant correlations with prognostic outcomes. Subsequently, to avoid overfitting and address severe multicollinearity, the glmnet package (v4.1-6) was used for Least Absolute Shrinkage and Selection Operator (LASSO) regression analysis. Multivariate Cox regression analysis was also conducted to assess the impact of multiple factors on patient survival.

The constructed prognostic model defined the individual risk score as Risk score = ∑_n_(X_i_ × Y_i_), where each coefficient (X_i_) was multiplied by the corresponding gene expression value (Y_i_). Utilizing the optimal cutoff generated by surv_cutpoint, patients were assigned to high- or low-risk categories in relation to bioA-associated gene expression patterns. Survival outcomes were analyzed with the “survival” R package (v3.3.1), and Kaplan–Meier survival curves were produced by ggsurvplot. Additionally, to quantify the model’s predictive accuracy, time-dependent ROC curves were constructed, and their associated AUC metrics were derived through the “TimeROC” package.

### Tumor microenvironment immune cell infiltration analysis and somatic mutation profiling

Glioma stromal, immune, and combined ESTIMATE scores were assessed and visualized with the ESTIMATE package (v1.0.13), while immune cell-associated scores were determined in the CIBERSORT package (v0.1.0). Additionally, to evaluate immune cell infiltration, the xCell package was used to identify potential immune cell subgroups and analyze their relative abundances. Tumor immune escape was assessed through the tumor immune dysfunction and exclusion(TIDE) tool, utilizing gene expression data derived from the analyzed tumor specimens. Tumor immune escape potential was assessed via the TIDE tool, with analysis predicated on the gene expression profiles of tumor samples.

The TCGA biolinks and maftools R packages were used to analyze and visualize mutation data, and the correlation between glioma-related DEGs and tumor mutation burden (TMB) was calculated.

### Drug sensitivity prediction

To predict clinical chemotherapy responses, drug half-maximal inhibitory concentration (IC50) values were calculated using the pRRophetic R package (v0.5) in conjunction with the GDSC database, with predictions anchored on baseline gene expression levels.

### Cell culture and reagents

LN229 and U251MG human glioma cell lines were sourced from the Cell Bank of the Chinese Academy of Sciences (Shanghai, China). Prior to use, all cell lines underwent short tandem repeat (STR) profiling for authentication and tested negative for mycoplasma. Cells were maintained in Dulbecco’s Modified Eagle Medium (DMEM) (Gibco, Cat#11965-092) containing 10% FBS (Gibco, Cat#10270-106) and 1% penicillin–streptomycin (Thermo Fisher Scientific, Cat#15140-122) under standard culture conditions (37 °C, 5% CO_2_, humidified atmosphere). Key reagents used in this research included the Cell Counting Kit-8 (CCK-8; Dojindo, Cat#CK04-500), Annexin V-FITC/PI apoptosis detection kit (BD Biosciences, Cat#556547), crystal violet (Sigma-Aldrich, Cat#C0775), and TRIzol reagent (Invitrogen, Cat#15596018) for total RNA isolation.

To investigate the role of oxidative stress in BioA-induced cellular responses, glioma cells were treated with BioA in the presence or absence of the antioxidant N-acetyl-L-cysteine (NAC). LN-229 and U251-MG cells were seeded in appropriate culture plates and allowed to attach overnight. BioA was dissolved in DMSO to prepare stock solutions, and the final DMSO concentration in culture medium was kept below 0.1%. Cells were then exposed to BioA (20 μM) for the indicated time periods. For ROS scavenging experiments, cells were pretreated with NAC (5 mM) for 1 h before BioA administration and maintained in NAC-containing medium during BioA exposure. Control cells received vehicle treatment. After treatment, cells were collected for subsequent analyses including ROS measurement, glutathione assays, colony formation assays, and cell viability assays.

### CRISPR/Cas9-mediated knockout of *GSTP1*

To explore the biological role of *GSTP1*, which was identified by single-cell transcriptomic analysis as a metabolic marker of malignant glioma subclusters, a CRISPR/Cas9-based knockout strategy was employed.

Two sgRNAs specific to exon 2 of the *GSTP1* gene were designed using CRISPOR and subcloned into the lentiCRISPR-v2 vector (Addgene plasmid #52961). The corresponding sequences were:

- sg-*GSTP1*#1: 5′-GGAGCTGTTGTTGTTGAGTG-3′.
- sg-*GSTP1*#2: 5′-CAGTGGAATACGACCTCTCA-3′.

Lentiviral particles were produced in HEK293T cells via co-transfection of lentiCRISPR-v2-sg*GSTP1* or sgCtrl together with psPAX2 and pMD2.G plasmids using Lipofectamine 3000 (Invitrogen, Cat#L3000-015). Viral supernatants were harvested 48 h after transfection, passed through a 0.45 µm filter, and subsequently applied to infect LN229 and U251MG cells with 8 µg/mL polybrene (Sigma, Cat#TR-1003-G). Puromycin at a concentration of 2 µg/mL was used for the selection of stable transductants, lasting 5–7 days. We confirmed the efficiency of *GSTP1* knockout through qRT-PCR and Western blot analyses.

### Quantitative real-time PCR

Total RNA was extracted with TRIzol reagent following the supplied protocol. Complementary DNA was generated using the PrimeScript RT reagent kit (Takara, Cat#RR037A). The qPCR was performed on a CFX96 Real-Time PCR Detection System (Bio-Rad, USA) using TB Green Premix Ex Taq II (Takara, Cat#RR820A) (56–59). Primer sequences were as follows:

GSTP1.

Forward: 5′-AGGACCTCCGCTTCTGAACT-3′.

Reverse: 5′-TCTCCCTCATCTACACCACT-3′.

SLC7A11.

Forward: 5′-CTGCTTTGGCTGTGTTGGTT-3′.

Reverse: 5′-AGGGTCTTCACCTTGATGCC-3′.

HMOX1 (HO-1).

Forward: 5′-AAGACTGCGTTCCTGCTCAA-3′.

Reverse: 5′-AAAGCCCTACAGCAACTGTCG-3′.

NQO1.

Forward: 5′-TGGGCAAGTCCATCCCAACT-3′.

Reverse: 5′-GCAAGTCAGGGAAGCCTGGA-3′.

GAPDH (reference gene).

Forward: 5′-GGAGCGAGATCCCTCCAAAAT-3′.

Reverse: 5′-GGCTGTTGTCATACTTCTCATGG-3′.

The 2^–ΔΔCt method was employed to calculate relative expression, using GAPDH as the internal control for normalization.

### Cell proliferation assay (CCK-8)

Cells (2 × 10³/well) were seeded in triplicate in 96-well plates. At 0–4 days following cell seeding, each well received 10 µL CCK-8 reagent, which was then incubated at 37 °C for 2 h. Absorbance at 450 nm was subsequently detected with a BioTek Synergy HTX microplate reader. The optical density (OD_450_) value was directly proportional to viable cell number, reflecting proliferation kinetics (60, 61).

### Colony formation assay

LN229 and U251MG cells were seeded at densities of 500–1000 cells per well in 6-well plates for long-term clonogenic survival assays. After 10–14 days of culture, formed colonies were fixed with 4% paraformaldehyde for 15 min, stained with 0.1% crystal violet for 20 min, and colonies comprising over 50 cells were enumerated under a stereomicroscope. Quantification of relative colony formation was performed with ImageJ.

### Wound-healing and transwell migration assays

Cells for the wound-healing assay were seeded into 6-well plates and allowed to grow to about 90% confluence. Linear scratches were made using a sterile 200 µL pipette tip to create a wound area. After removing floating cells with two PBS washes, the adherent cells were maintained in serum-free medium for 72 hours. Images were captured at 0 h, 24 h, 48 h, and 72 h using an inverted microscope (Leica DMi8), and wound closure rates were calculated using ImageJ. In the Transwell assay, 2 × 10^4^In the Transwell assay, 2 cells in serum-free medium were seeded into the upper chamber of 8 µm-pore inserts (Corning, Cat#3422), with 10% FBS-containing medium in the lower chamber to serve as a chemoattractant. At 24 h post-incubation, migrated cells were fixed and stained with 0.1% crystal violet and enumerated across five random fields under 200× magnification.

### Apoptosis analysis by flow cytometry

Cell apoptosis was evaluated with the Annexin V-FITC/PI kit (BD Biosciences, Cat#556547). A total of 1 × 10^6^ cells were harvested, washed twice in cold PBS, and resuspended in 100 µL binding buffer containing 5 µL Annexin V-FITC and 5 µL propidium iodide. After 15 minutes of incubation in the dark at room temperature, fluorescence signals were detected on a BD FACSCanto II flow cytometer. Data analysis was performed using FlowJo v10, which quantified early (Annexin V^+^/PI^-^) and late (Annexin V^+^/PI^+^) apoptotic populations.

### Intracellular ROS measurement

Intracellular reactive oxygen species (ROS) levels were measured using the DCFH-DA fluorescent probe (Beyotime, Shanghai, China) according to the manufacturer’s instructions. Briefly, treated cells were incubated with 10 μM DCFH-DA at 37 °C for 20 min in the dark. After incubation, cells were washed three times with serum-free medium to remove excess probe. The fluorescence intensity was then quantified using a fluorescence microplate reader (Ex 488 nm). ROS levels were normalized to the untreated sg-Ctrl group, and results were presented as relative ROS levels.

### Measurement of intracellular GSH/GSSG ratio

The intracellular GSH/GSSG ratio was determined using a commercial glutathione assay kit (Beyotime, Shanghai, China) following the manufacturer’s instructions. Briefly, cells were harvested and lysed, and total glutathione and oxidized glutathione (GSSG) were measured using an enzymatic recycling method. Reduced glutathione (GSH) levels were calculated according to the kit protocol, and the GSH/GSSG ratio was subsequently determined. The data were normalized to the untreated sg-Ctrl group and presented as relative values.

### Statistical analysis

All statistical analyses were carried out using R software (v4.3.0), Python (v4.2.0).All experiments were independently repeated at least three times. Data are presented as mean ± standard deviation (SD). Statistical significance was evaluated using a two-tailed Student’s t-test for two-group comparisons or one-way ANOVA followed by Tukey’s *post-hoc* test for multiple comparisons, using GraphPad Prism 9.0 software. Statistical significance was defined as p < 0.05.

## Results

### Glioma large group identification

**Figure 1:**
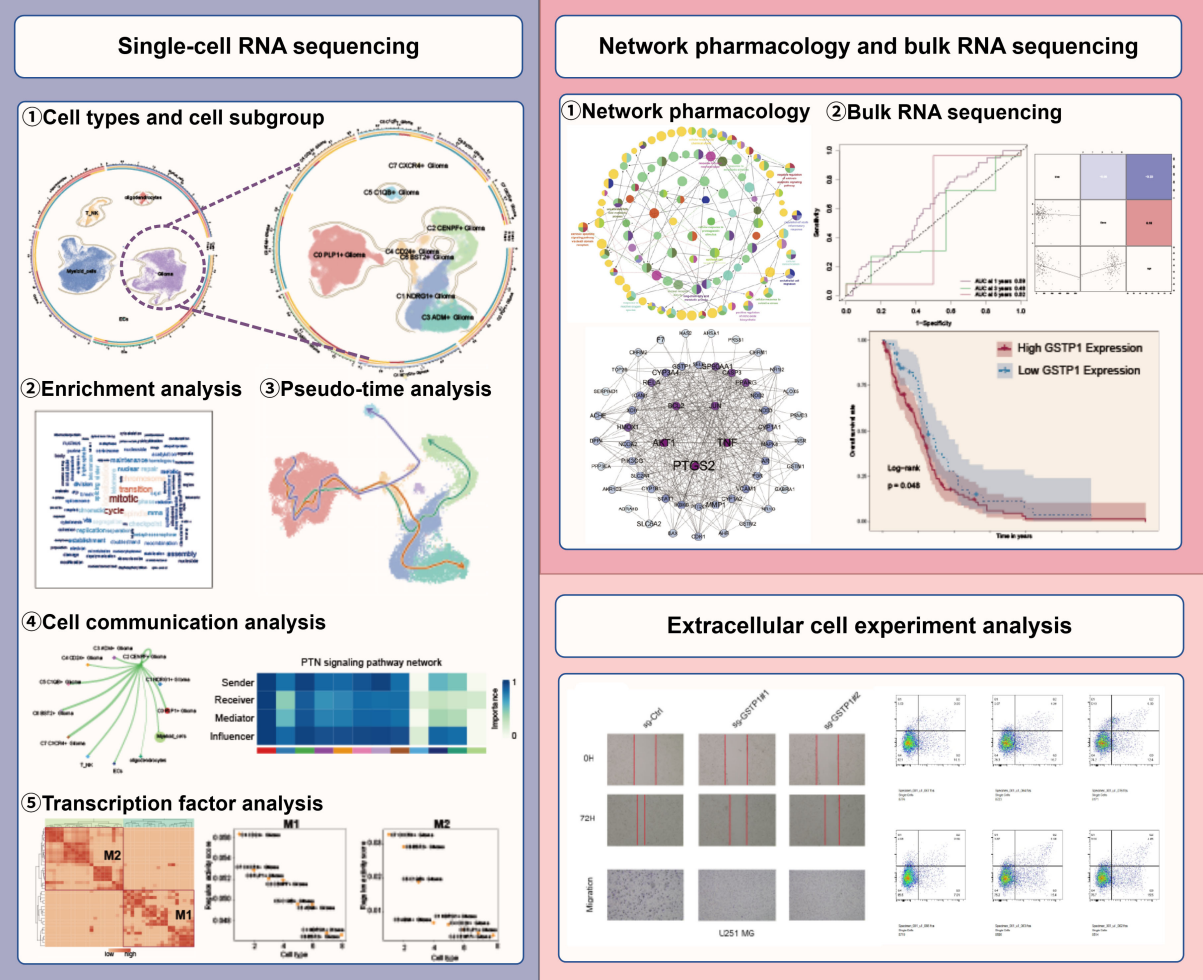
Flow chart.

**Figure 2:**
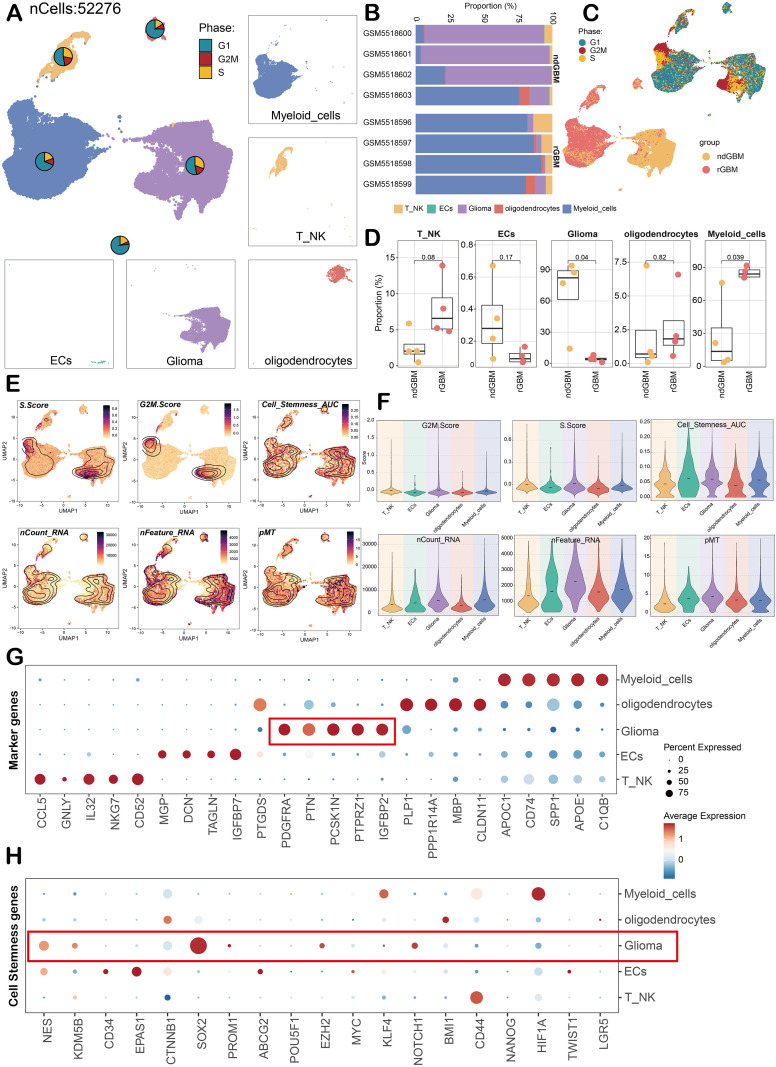
Single-cell atlas of glioma. **(A)** The UMAP map and pie chart showed the distribution and phase ratio of five cell types in glioma, including myeloid cells, T NK, oligodendrocytes, glioma, and ECs. The surrounding faceted map visualizes the distribution of the five cells. **(B)** The stacked bar graph showed the proportion of five kinds of cells in different groups and samples. **(C, D)**. UMAP and box plots showed the cell cycle and group distribution of the five cells. **(E, F)**. The G2M Score, Score, Cell *Stemness* AUC, nCount RNA, nFeature RNA, and pMT of the five cells were visualized by UMAP and violin plots. **(G, H)**. Bubble diagram showed the expression levels of key marker genes and stemness genes in five cell types. The size of the bubble corresponds to the percentage of gene expression, and the color represents the average expression.

Firstly, we presented the research sequence through a flowchart (**Figure 1**). In order to explore the cell heterogeneity of glioblastoma (GBM), this study comprehensively analyzed the scRNA-seq data of patients with new glioma (ndGBM) and recurrent glioma (rGBM). After quality control, the cells were divided into five main cell groups by the dimensionality reduction clustering method, namely myeloid cells, T/NK (T and NK cells), oligodendrocytes, glioma, and endothelial cells (ECs), and the cell cycle distribution characteristics of each cell group (**Figure 2A**) were presented by charts. The large group contains 52,276 cells. The bar charts showed that glioma cells accounted for a significantly higher proportion in ndGBM, while myeloid cells were significantly more prevalent in rGBM (**Figure 2B**). The UMAP diagram further demonstrated the distribution relationship between phase and group. It could be seen that glioma was mainly concentrated in the G2/M phase and S phase (**Figure 2C**). The box plots intuitively showed the specific percentage difference (**Figure 2D**) of various cells in ndGBM and rGBM. To conduct analysis of the functional characteristics of each cell type, UMAP and violin plots were used in this study to present the S Score, G2/M Score, Cell Stemness AUC, nCount RNA, nFeature RNA, and pMT of cell populations. These revealed that glioma cells exhibited significantly elevated scores for the aforementioned indicators (**Figures 2E, F**). We further identified the marker genes for each cell type through the use of bubble diagrams. The key genes of glioma were *PDGFRA, PTN, PCSK1N, PTPRZ1*, and *IGFBP2*, and the core stem genes were *NES, SOX2*, and *NOTCH1* (**Figures 2G, H**). Additionally, Ro/e analysis results further confirmed that glioma cells were highlyexpressed in the G2/M and S phases (**Supplementary Figure 1A**). To explore the biological function differences among distinct cell types, the DEGs (**Supplementary Figure 1B**) of myeloid cells, oligodendrocytes, glioma cells, endothelial cells, and T/NK cells werescreened by volcano plot. Word cloud analysis revealed that the function of glioma cells is primarily associated with localization (**Supplementary Figure 1C**). Glioma showed significantly positive enrichment in axon development, oligodendrocytedifferentiation, mitochondrial ATP synthesis coupled with electron transport, glial cell differentiation, and so on (**Supplementary Figure 1D**).

### Identification of tumor cell subgroups

**Figure 3:**
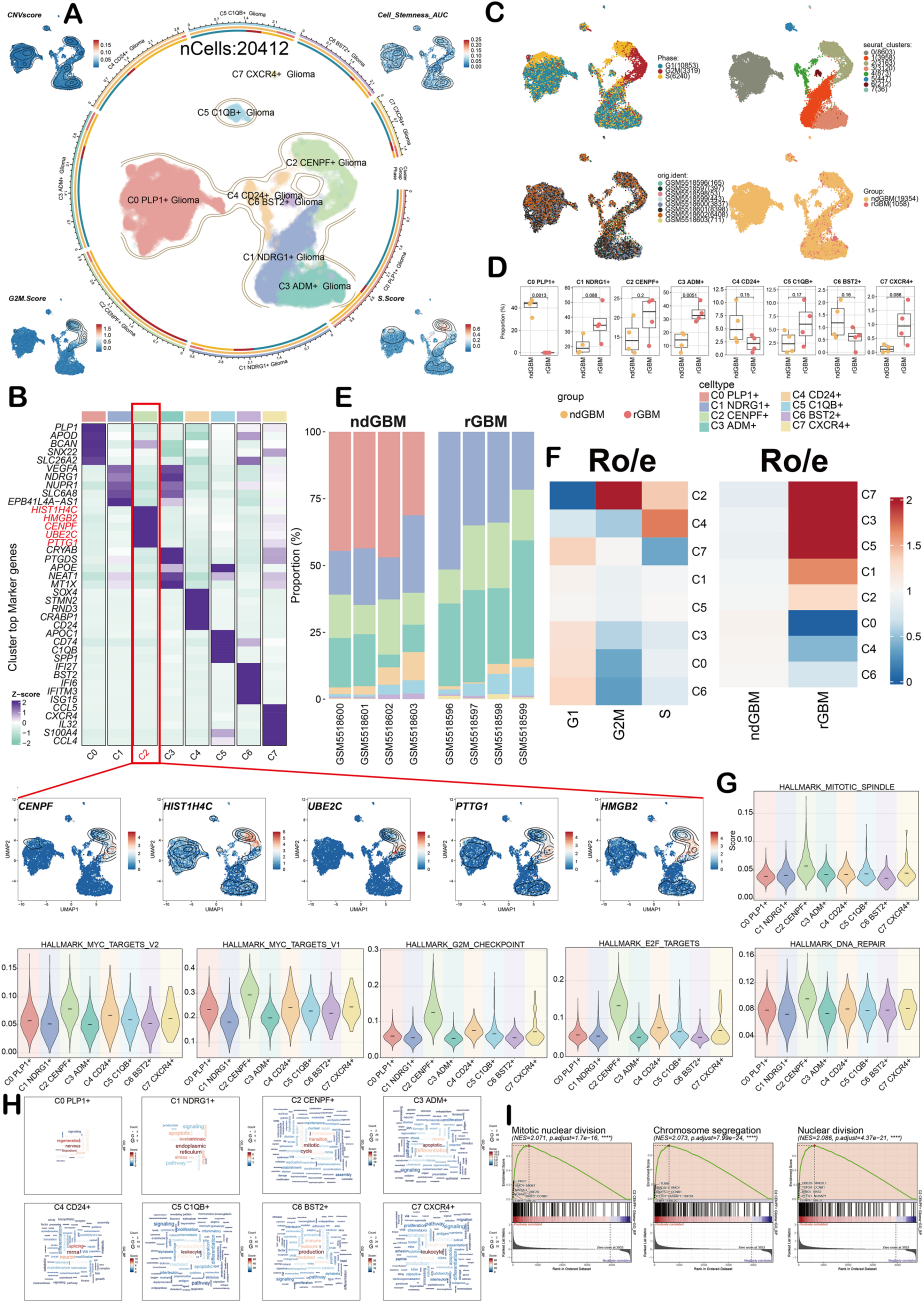
Single-cell atlas of glioma cell subgroups. **(A)** The circular graph showed the clustering of eight cell subgroups identified in glioma, and the contour curve outlines the boundaries of each cell subgroup. The peripheral coordinate axis displayed the total number of cells in each category with a logarithmic scale. The UMAP map was located at the four corners, and the CNV score, cell stemness AUC, S score, and G2/M score were presented clockwise from the upper left corner. **(B)** A heat map showed the expression of cluster top marker genes in eight cell subgroups. Marker genes belong to the C2 subgroup, including *CENPF, UBE2C, PTTG1, HIST1H4C*, and *HMGB2*. UMAP technology was used to visualize the marker genes of the C2 subgroup. **(C)** The UMAP maps of phase (S phase, G2/M phase, and G1 phase), Seurat clusters (0-7), orig.ident (GSM5518596-GSM5518603), and group (ndGBM, rGBM) in glioma cell subgroups. **(D)** The box plot showed the proportion of 8 glioma cell subgroups in ndGBM and rGBM. **(E)** The bar chart showed the expression percentage of eight cell subgroups in each sample of ndGBM and rGBM. **(F)** Ro/e score was used to evaluate the tissue preference of eight cell subgroups to different phases and groups. **(G)** Violin plot showed the value of eight cell subgroups in multiple pathways. **(H)** The word cloud map showed the expression of key functions in eight cell subgroups. **(I)** GSEA plots showed the up-regulated enrichment pathway of the C2 subgroup.

In order to further analyze the heterogeneity of glioma, this study used the inferCNV tool. The results exhibited that the glioma cell population had obvious malignant cell characteristics (**Supplementary Figure 2**). Based on the above analysis, the tumor cells were reclassified, and combined with the marker gene annotation of each cell, eight glioma cell subgroups were finally identified, which were C0 *PLP1*^+^ Glioma, C1 *NDRG1*^+^ Glioma, C2 *CENPF* ^+^ Glioma, C3 *ADM* ^+^ Glioma, C4 *CD24* ^+^ Glioma, C5 *C1QB* ^+^ Glioma, C6 *BST2* ^+^ Glioma, and C7 *CXCR4* ^+^ Glioma. The CNV score, G2/M score, S score, and cell stemness AUC (**Figure 3A**) of each subgroup were displayed by UMAP. The subgroup contained a total of 20,412 cells. The heatmap results showed the key differential genes of each glioma subgroup, among which the core genes of the C2 subgroup included *HIST1H4C, HMGB2, CENPF, UBE2C*, and *PTTG1* (**Figure 3B**), and the distribution of these key genes was visually presented by UMAP. At the same time, UMAP analysis was performed in combination with cell cycle (G1, G2M, S phase), sample source (GSM5518596-GSM5518603), clustering results (0–7), and grouping information (ndGBM, rGBM). Then, the C2 subgroup was predominantly distributed in the G2/M and S phases (**Figure 3C**). The box plot analysis distributed that the proportion of the C0 subgroup in ndGBM was higher, while the proportion of the C1 subgroup and the C2 subgroup in rGBM was increased (**Figure 3D**). Bar chart analysis further confirmed that the proportion of the C2 subgroup was higher in rGBM (**Figure 3E**). The heatmap plot showed that the Ro/e value of the C2 subgroup was the highest in the G2M and S phases, suggesting that this subgroup may be closely related to tumor progression (**Figure 3F**). Further verified by violin plot analysis, the C2 subgroup showed significantly elevated expression in multiple pathways including MITOTIC SPINDLE, MYC TARGETS V2/V1, G2M CHECKPOINT, E2F TARGETS, and DNA REPAIR, and these pathways are critical for promoting tumor progression. It was indicated that the C2 subgroup is one of the core subgroups (**Figure 3G**) that affect the progression of glioma. To clarify the function of the C2 subgroup, it was found that it was enriched in mitosis, cell cycle, cell transition, and other related pathways (**Figure 3H**) by word cloud plots. Further GSEA demonstrated that the C2 subgroup was significantly upregulated in the pathways of mitotic nuclear division, chromosome segregation, and so on (**Figure 3I**).

### Pseudotime analysis

**Figure 4:**
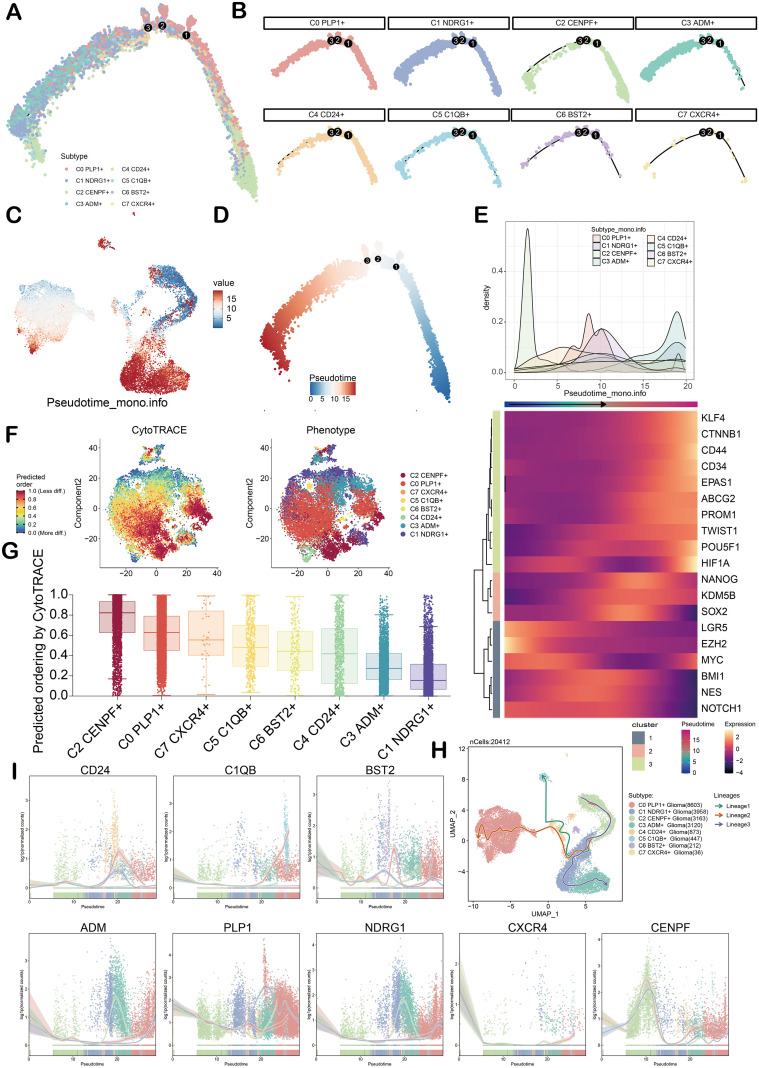
Pseudotime analysis of glioma cell subgroups. **(A)** Monocle trajectory showed the pseudo-time series analysis of glioma cell subgroups. **(B)** The monocle trajectory showed the pseudo-time series analysis of each glioma cell subgroup. **(C)** UMAP diagrams showed the value of pseudotime. **(D)** Monocle trajectory showed the pseudo-time series analysis of glioma cell subgroups. **(E)** The combination of ridge map and heat map showed the dynamic expression of stemness genes in each glioma cell subgroup in the whole quasi-sequential trajectory. **(F)** The left image showed the distribution of genes at different differentiation stages over time, in which the color depth represents the level of cell stemness. The distribution of glioma cell subgroups is shown on the right side, and each subgroup is labeled with different colors. **(G)** CytoTRACE analysis was used to rank the predicted stemness of glioma cell subgroups. **(H)** The UMAP diagram showed the differentiation trajectory of each glioma cell subgroup (lineage1-3), with green representing lineage 1, red representing lineage2, and blue representing lineage3. **(I)** The dynamic trend diagram showed the dynamic expression of the named genes of each glioma subgroup in lineage1-3.

To clarify the differentiation trajectory of each glioma subgroup, this study integrated Monocle, Cytotrace, and Slingshot tools for pseudotime series analysis. Firstly, the UMAP demonstrated the pseudotime trajectory of glioma subgroups. In general, all cell subgroups showed a developmental trajectory from the lower right corner to the upper left corner and then changed to the lower left corner. Notably, the C2 subgroup was distributed at both the initial and terminal points of the pseudotime trajectory (**Figures 4A–D**). Through the analysis of the ridge map and heatmap, the dynamic expression pattern of stem genes in each glioma subgroup in the whole pseudotime trajectory was presented. The results showed that the genes such as *LGR5* in the C2 subgroup were highly expressed (**Figure 4E**). Cytotrace analysis showed that the predicted stemness level of the C2 subgroup was the highest Using the Slingshot tool to construct the pseudotime trajectory of glioma subgroups, three differentiation lineages (lineage1, lineage2, and lineage3) were identified (**Figures 4F, G**). UMAP map showed that lineage1–3 originated from C2 subgroup (**Figure 4H**). The expression patterns of genes for the various glioma subgroups were further illustrated in lineage 1-3 (**Figure 4I**).

### CellChat analysis and TFs distribution

**Figure 5:**
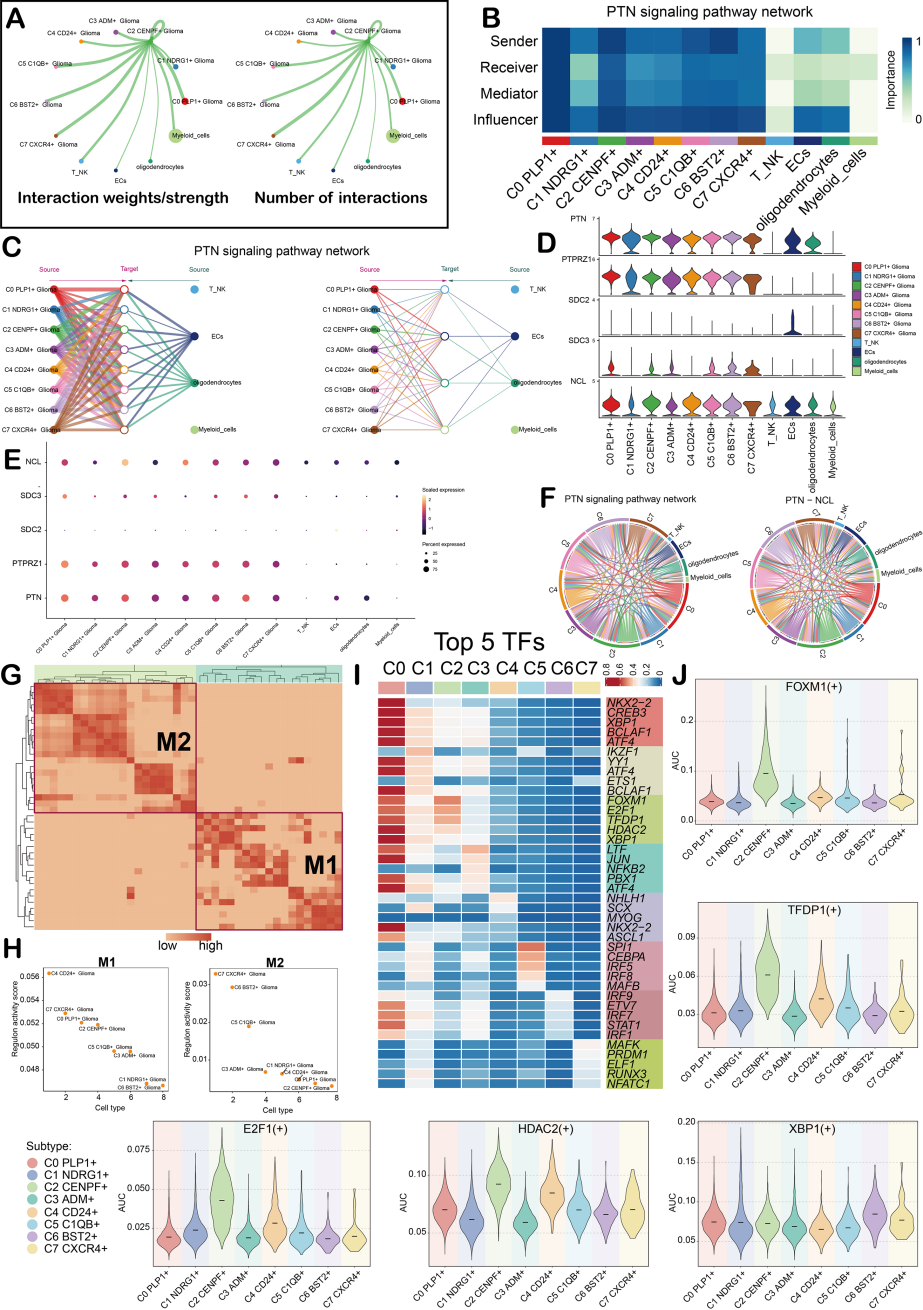
Cell communication and TFs between the C2 subgroup and other cell types. **(A)** Circle plot showed the interaction weights/strength and number of interactions between the C2 subgroup and other cell types. **(B)** The heatmap showed the centrality score map of the PTN signaling pathway. In addition, the importance scores of each glioma cell subgroup in sender, receiver, mediator, and influencer were also shown. **(C)** The hierarchical diagram showed the interaction between C2 subgroup and other cell types in the PTN signaling pathway. **(D, E)**. The violin plot **(D)** and bubble plot **(E)** showed the relationship between cell types and related genes. The C2 subgroup and other cell types may interact with each other through ligand PTN and receptor NCL. **(F)** The circle diagram showed the interaction between various cell types in the PTN signaling pathway and the PTN-NCL ligand-receptor pair. **(G)** The heatmap showed the regulatory modules identified in glioma cells, including M1 and M2. **(H)** The scatter plot showed the regulon activity scores of glioma cell subgroups in M1 and M2. **(I)** The heatmap showed the top five TFs in each glioma cell subgroup. **(J)** Violin plot showed the AUC of the top five TFs in the C2 subgroup in each glioma subgroup.

Explore the communication mechanism between cells in the glioma microenvironment, this study constructed a cell communication network between the C2 subgroup and other cell types through the CellChat tool (**Figure 5A**). The signaling pathway network indicated that the C2 subgroup mainly engages in interactions with other cell types through the PTN signaling pathway. Among them, the C2 subgroup mainly acted as a ‘sender,’ while ECs also served as a ‘sender,’ ‘receiver,’ and ‘influencer.’ (**Figure 5B**). The specific interaction between the C2 subgroup and ECs or oligodendrocytes (**Figure 5C**) was further demonstrated by the hierarchical diagram. The expression profiles of core ligand-receptor pairs within the PTN signaling pathway were visualized. It was observed that tumor cell subgroups showed high PTN expression, with NCL being highly expressed in all cell types (**Figures 5D, E****).** The circle diagram clearly showed the PTN pathway and the interaction network between cells in the PTN-NCL ligand-receptor pair (**Figure 5F**). To analyze the regulatory role of TFs in glioma subgroups, TFs with similar functions and expression patterns were first divided into two modules (M1, M2), and the distribution characteristics of these two modules were demonstrated by UMAP diagram (**Supplementary Figure 3A**; **Figures 5G, H**). Sorting the regulon activity scores of TFs in M1 and M2 modules demonstrated that the C2 subgroup had a more notable correlation with the M1 module (**Figure 5I**). The heatmap results showed the top 5 TFs in glioma subgroups. The core TFs of the C2 subgroup were FOXM1, E2F1, TFDP1, HDAC2, and XBP1 (**Figure 5J**). The scatter plot (**Supplementary Figure 3B**) and the violin plot (**Figure 5L**) further verified the high expression characteristics of these core TFs in the C2 subgroup (represented by AUC values).

### BioA related genes and enrichment analysis

**Figure 6:**
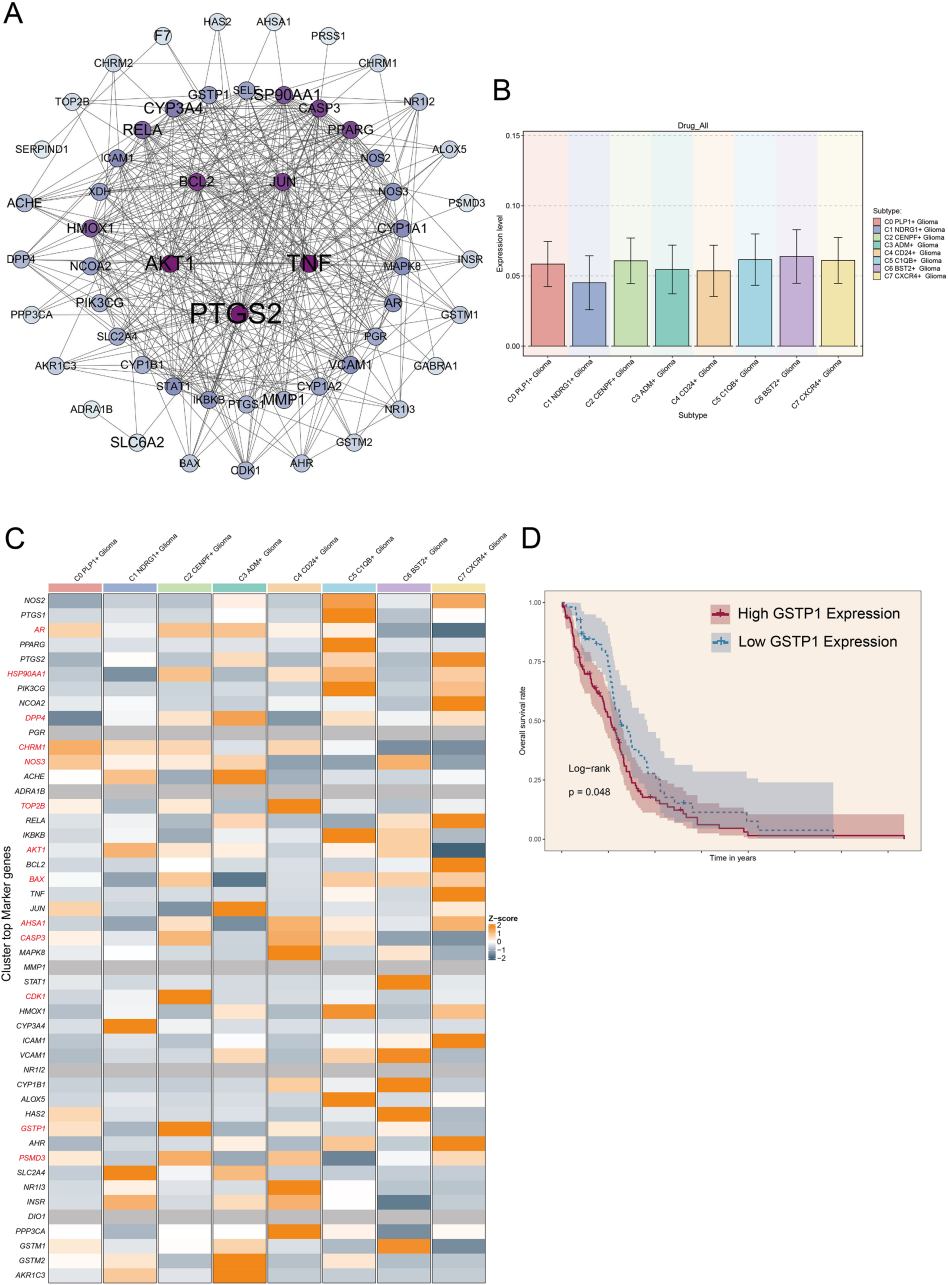
BioA-related genes **(A)** The PPI diagram showed that the genes are significantly related to bioA. The larger the point, the stronger the significance, and the more purple the color, the stronger the significance. **(B)** A bar plot showed the tumor cell subgroups and group scores in the bioA pathway. **(C)** The heatmap showed the expression of bioA-related genes in each tumor cell subgroup. **(D)** The KM survival curve showed the prognosis of the high and low *GSTP1* groups.

The network diagram analysis showed the drug-gene interaction relationship related to active Chinese medicine component bioA, among which the genes most closely related to bioA included *PTGS2, AKT1, TNF, BCL2, JUN, SP90AA1, GSTP1*, etc. (**Figure 6A**). Through bioA pathway analysis, the C2 subgroup was found to have the highest score in this pathway, confirming its strong association with the bioA pathway (**Figure 6B**). By collecting bioA-related genes and performing expression analysis, it was found that a variety of drug-related genes were highly correlated in the C2 subgroup, including *AR, HSP90AA1, DPP4, CHRM1, NOS3, TOP2B, AKT1, BAX, AHSA1, CASP3, CDK1, GSTP1, PSMD3*, etc. (**Figure 6C**). The Kaplan–Meier survival curve indicated that patients exhibited *GSTP1* levels had significantly worse survival outcomes (**Figure 6D**).

GSEA demonstrated that bioA-related genes were mainly enriched in cellular response to prostaglandin stimulus, epithelial cell apoptotic process, response to ROS, and other pathways (**Supplementary Figure 4**). It was worth noting that the bioA pathway was enriched with many pathways related to ROS, which was consistent with the characteristics of *GSTP1* as a highly related gene in the bioA pathway.

### *GSTP1* knockout inhibits glioma cell proliferation and migration while promoting apoptosis

Based on our single-cell transcriptomic analysis, *GSTP1* was predominantly enriched in the malignant glioma subclusters characterized by high oxidative stress response and cell cycle activity. To experimentally validate its functional role, we performed loss-of-function assays using two independent sgRNAs targeting *GSTP1* in LN229 and U251MG glioma cells.

**Figure 7:**
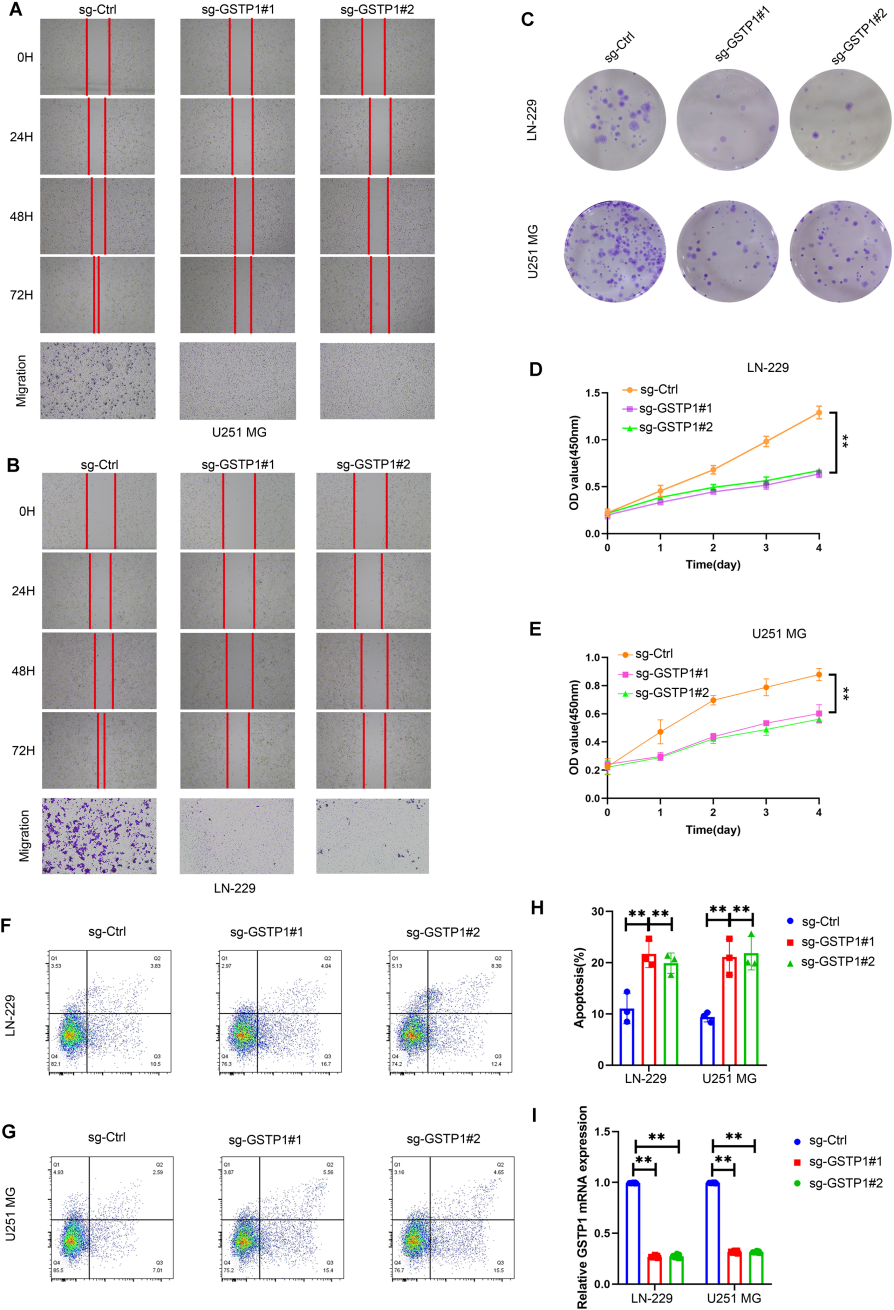
*GSTP1* knockout suppresses proliferation and migration while inducing apoptosis in glioma cells. **(A, B)** Wound healing and Transwell migration assays of U251MG and LN229 cells transduced with sgCtrl or two independent sgRNAs targeting *GSTP1* (sg-*GSTP1*#1 and sg-*GSTP1*#2). Representative images at 0 h, 24 h, 48 h, and 72 h were shown; lower panels depict crystal violet-stained migrated cells (scale bar = 100 μm). **(C)** Colony formation assay showing reduced clonogenic growth after *GSTP1* knockout in both cell lines. **(D, E)** CCK-8 proliferation curves of LN229 and U251MG cells showing significantly lower OD450 values in sg-*GSTP1* groups compared with sgCtrl (mean ± SD, n = 3; **p < 0.01. **(F, G)** Flow cytometric detection of apoptosis using Annexin V–FITC/PI staining in LN229 and U251MG cells following *GSTP1* depletion. **(H)** Quantification of apoptotic cell percentages from **(F, G)**. **(I)** The qRT-PCR validation of *GSTP1* mRNA downregulation in sg-*GSTP1*#1 and sg-*GSTP1*#2 groups relative to sgCtrl. Data are presented as mean ± SD; **p < 0.01 (Student’s t-test).

As shown in **Figures 7A, B**, wound-healing assays performed at sequential time points (0, 24, 48, and 72 h) revealed a progressive inhibition of migratory capacity following GSTP1 depletion in both LN229 and U251MG glioma cells. Compared with sgCtrl cells, which displayed rapid wound closure over time, GSTP1-deficient cells exhibited a markedly delayed closure of the scratch gap beginning at 24 h and persisting through 48 h and 72 h. Consistent with the wound-healing results, Transwell migration assays demonstrated a substantial reduction in the number of migrated cells following GSTP1 knockout. These findings collectively indicate that GSTP1 plays an important role in sustaining the migratory phenotype of glioma cells. Clonogenic and CCK-8 proliferation assays further demonstrated that *GSTP1* depletion significantly inhibited long-term colony formation and cell growth (**Figures 7C–E**). The growth curves indicated a progressive reduction in cell viability over four days following *GSTP1* knockout, consistent across both cell lines, suggesting a growth-suppressive effect of *GSTP1* silencing.

Flow cytometric analysis of Annexin V–FITC/PI staining (**Figures 7F–H**) demonstrated a substantial increase in apoptotic cell fractions in sg-*GSTP1*#1 and sg-*GSTP1*#2 groups compared to sgCtrl, indicating that *GSTP1* loss promotes apoptosis in glioma cells. Finally, qRT-PCR confirmed effective downregulation of *GSTP1* mRNA expression in both cell lines (**Figure 7I**), verifying the efficiency of CRISPR-mediated gene knockout.

The experiment confirmed that *GSTP1* promoted glioma cell proliferation and migration while protecting cells from apoptosis, supporting the single-cell findings that *GSTP1*-high malignant clusters possess enhanced metabolic resilience and proliferative potential through oxidative stress adaptation mechanisms.

### BioA induces ROS-dependent oxidative stress and suppresses glioma cell proliferation

**Figure 8:**
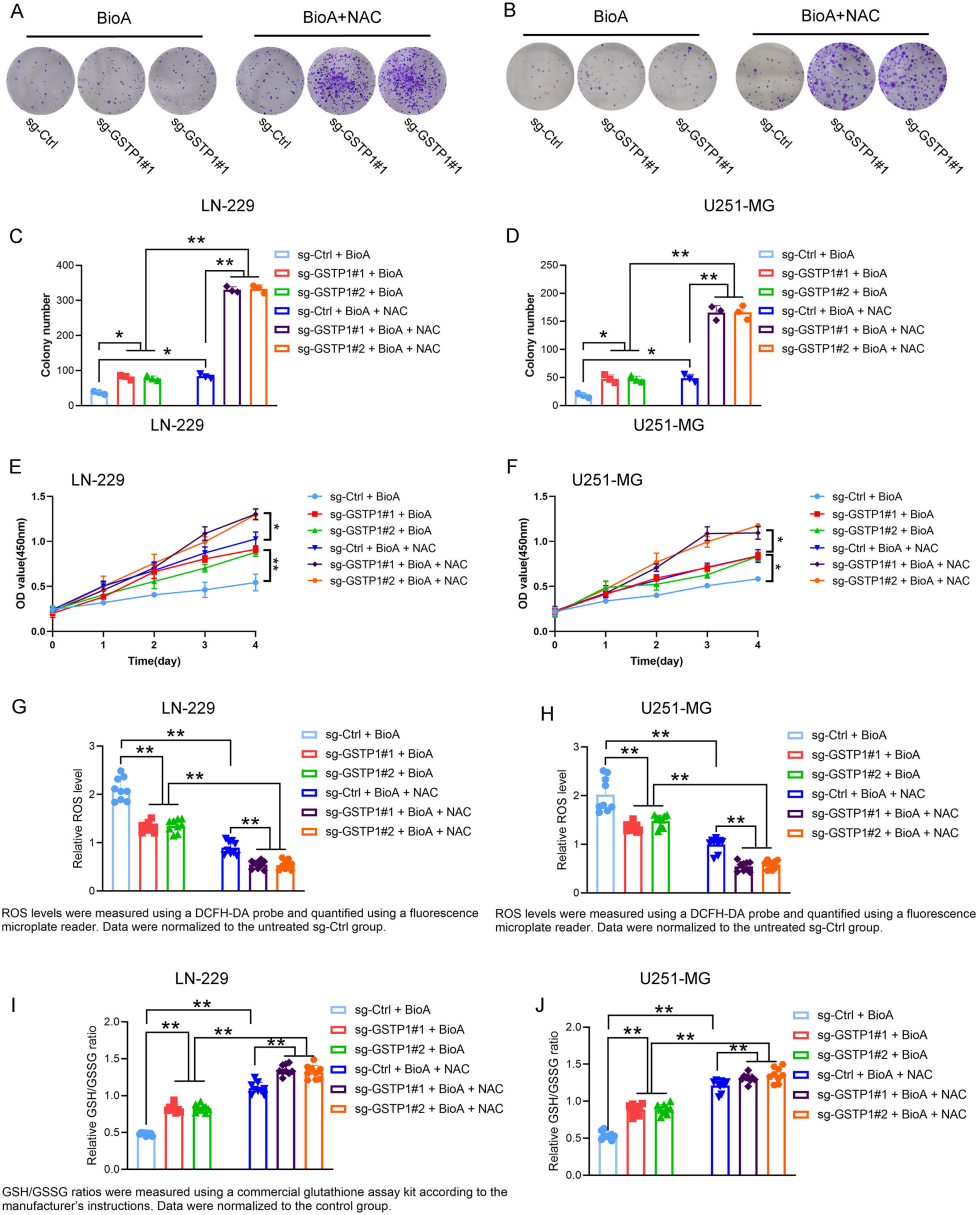
BioA induces oxidative stress and suppresses glioma cell proliferation in a ROS-dependent manner. **(A, B)** Representative images of colony formation in LN-229 and U251-MG cells treated with BioA in the presence or absence of the ROS scavenger NAC. Cells were transduced with control sgRNA (sg-Ctrl) or GSTP1-targeting sgRNAs (sg-GSTP1#1 and sg-GSTP1#2) and subjected to the indicated treatments. **(C, D)** Quantification of colony formation in LN-229 and U251-MG cells under the indicated conditions. **(E, F)** Cell viability of LN-229 and U251-MG cells following the indicated treatments, determined using the CCK-8 assay. **(G, H)** Intracellular ROS levels in LN-229 and U251-MG cells treated with BioA with or without GSTP1 knockdown and NAC. ROS levels were measured using a DCFH-DA fluorescent probe and quantified by a fluorescence microplate reader. Data were normalized to the untreated sg-Ctrl group. **(I, J)** Relative GSH/GSSG ratios in LN-229 and U251-MG cells under the indicated conditions. GSH/GSSG ratios were measured using a commercial glutathione assay kit according to the manufacturer’s instructions. Data were presented as mean ± SD from three independent experiments. Statistical significance was determined using one-way ANOVA followed by multiple comparison tests. *p < 0.05, **P < 0.01.

To determine whether oxidative stress contributes to the antitumor effects of BioA in glioma cells, we first assessed intracellular ROS levels following BioA treatment in the presence or absence of GSTP1 depletion and ROS scavenging. As shown in **Figures 8G, H**, BioA treatment markedly increased intracellular ROS accumulation in both LN-229 and U251-MG cells. However, GSTP1 knockdown significantly attenuated BioA-induced ROS generation, as evidenced by the reduced ROS levels observed in the sg-GSTP1#1 + BioA and sg-GSTP1#2 + BioA groups compared with the sg-Ctrl + BioA group. Importantly, treatment with the antioxidant NAC effectively suppressed ROS accumulation across all groups, indicating that the increase in intracellular ROS induced by BioA is ROS-dependent and can be neutralized by antioxidant intervention.

To further evaluate the redox status of glioma cells under these conditions, we measured the intracellular GSH/GSSG ratio, a well-established indicator of oxidative stress and cellular redox homeostasis. As shown in **Figures 8I, J**, BioA treatment resulted in a marked decrease in the GSH/GSSG ratio in control cells, consistent with elevated oxidative stress. In contrast, GSTP1 depletion partially restored the GSH/GSSG ratio, suggesting that the reduction of GSTP1 alleviates BioA-induced redox imbalance. Consistent with the ROS data, NAC treatment further increased the GSH/GSSG ratio in all groups, indicating effective scavenging of intracellular ROS and restoration of cellular redox balance.

Given that oxidative stress can profoundly influence tumor cell growth, we next examined the functional consequences of ROS modulation on glioma cell proliferation. Colony formation assays revealed that BioA treatment significantly inhibited the clonogenic capacity of LN-229 and U251-MG cells (**Figures 8A–D**). Notably, GSTP1 knockdown further enhanced the suppressive effect of BioA on colony formation, suggesting that GSTP1 depletion sensitizes glioma cells to BioA-induced growth inhibition. However, the addition of NAC partially rescued colony formation ability, indicating that ROS generation contributes to the inhibitory effect of BioA on glioma cell proliferation.

Similarly, CCK-8 assays demonstrated that BioA treatment significantly reduced the viability of both LN-229 and U251-MG cells (**Figures 8E, F**). Consistent with the clonogenic assay results, GSTP1 depletion further decreased cell viability following BioA treatment, whereas NAC treatment partially restored cell viability. These findings indicate that ROS accumulation plays a critical role in mediating BioA-induced growth suppression in glioma cells.

Collectively, these results demonstrate that BioA induces oxidative stress in glioma cells, characterized by increased ROS production and disruption of glutathione redox balance. GSTP1 depletion attenuates ROS accumulation and partially restores cellular redox homeostasis, whereas antioxidant treatment with NAC reverses BioA-induced oxidative stress and growth inhibition. These findings suggest that GSTP1 participates in the regulation of BioA-induced oxidative stress signaling and redox homeostasis in glioma cells, thereby influencing tumor cell survival and proliferation.

### GSTP1 regulates BioA-induced transcriptional activation of oxidative stress–responsive genes

To further elucidate whether GSTP1 regulates cellular redox homeostasis in response to BioA treatment, we next examined the transcriptional profiles of several canonical oxidative stress–responsive genes, including SLC7A11, HMOX1, and NQO1, in glioma cells. These genes are well-established downstream effectors involved in antioxidant defense and glutathione metabolism and therefore serve as molecular indicators of intracellular oxidative stress.

**Figure 9:**
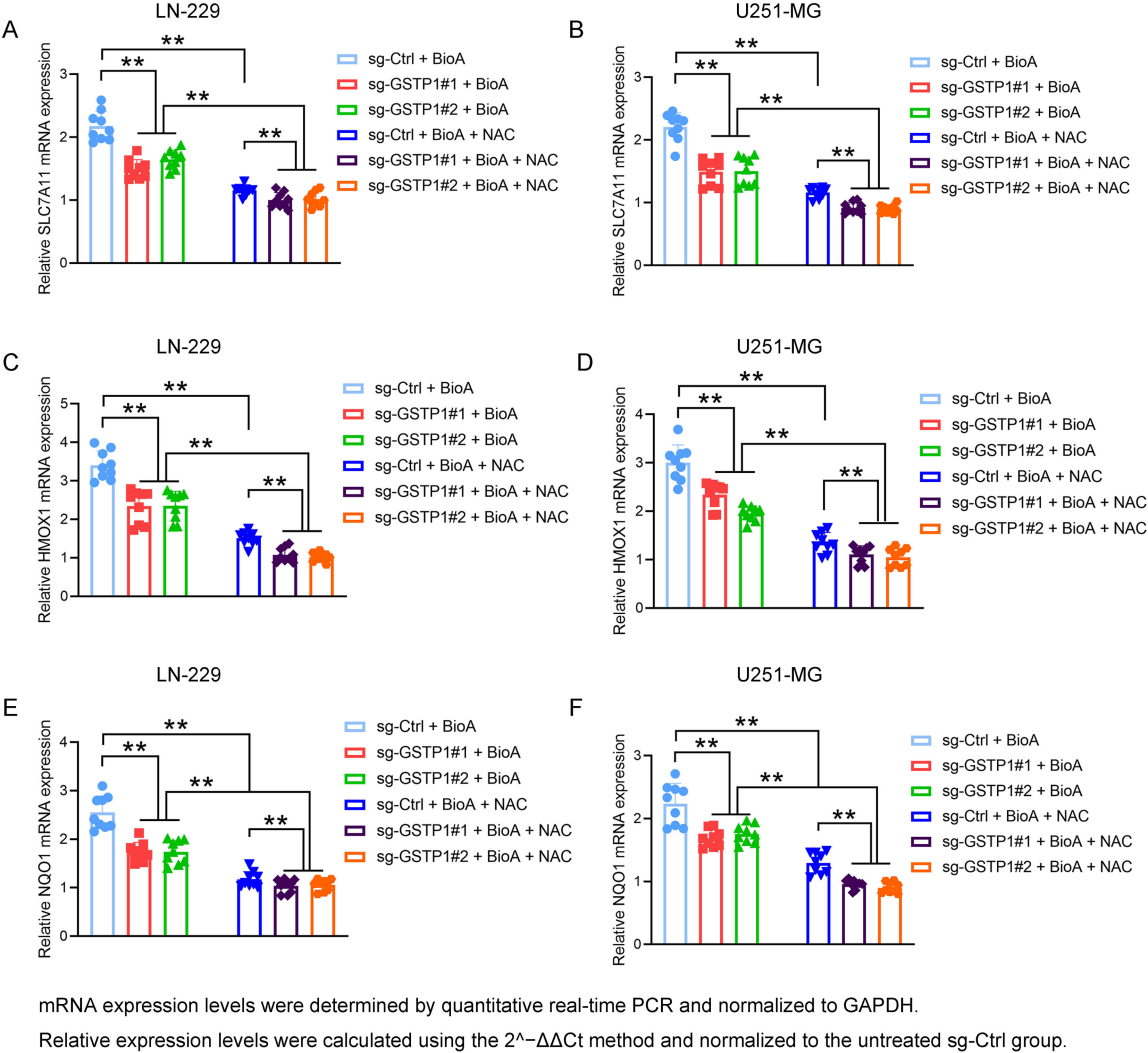
GSTP1 depletion attenuates BioA-induced transcriptional activation of oxidative stress–responsive genes in glioma cells. **(A, B)** Relative mRNA expression levels of SLC7A11 in LN-229 and U251-MG cells following BioA treatment in the presence or absence of GSTP1 knockdown and NAC. **(C, D)** Relative mRNA expression levels of HMOX1 in LN-229 and U251-MG cells under the indicated conditions. **(E, F)** Relative mRNA expression levels of NQO1 in LN-229 and U251-MG cells following the indicated treatments. Cells were transduced with control sgRNA (sg-Ctrl) or GSTP1-targeting sgRNAs (sg-GSTP1#1 and sg-GSTP1#2) and subsequently treated with BioA with or without the ROS scavenger NAC. mRNA expression levels were determined by quantitative real-time PCR and normalized to GAPDH. Relative expression levels were calculated using the 2^-ΔΔCt method and normalized to the untreated sg-Ctrl group. Data are presented as mean ± SD from three independent experiments. Statistical significance was determined using one-way ANOVA followed by multiple comparison tests. **P < 0.01.

Quantitative real-time PCR analysis demonstrated that BioA treatment markedly induced the expression of SLC7A11 in control glioma cells (sg-Ctrl + BioA) in both LN-229 and U251-MG cell lines (**Figures 9A, B**). In contrast, GSTP1 depletion significantly attenuated this induction, as evidenced by the reduced SLC7A11 mRNA levels observed in the sg-GSTP1#1 + BioA and sg-GSTP1#2 + BioA groups compared with the sg-Ctrl + BioA group. These findings indicate that GSTP1 contributes to BioA-induced activation of antioxidant transcriptional responses. Moreover, treatment with the ROS scavenger NAC further suppressed SLC7A11 expression across all groups, suggesting that the transcriptional upregulation of SLC7A11 is largely dependent on intracellular ROS accumulation.

Consistent with this observation, the expression of HMOX1, another classical oxidative stress–responsive gene, exhibited a similar pattern (**Figures 9C, D**). BioA treatment significantly elevated HMOX1 mRNA levels in control cells, whereas GSTP1 knockdown partially abrogated this BioA-induced transcriptional activation. Importantly, the addition of NAC markedly diminished HMOX1 expression, further supporting the notion that BioA triggers oxidative stress signaling in a ROS-dependent manner and that GSTP1 participates in modulating this response.

A comparable regulatory pattern was also observed for NQO1, a key antioxidant enzyme involved in detoxification and cellular redox regulation (**Figures 9E, F**). BioA treatment strongly induced NQO1 transcription in sg-Ctrl cells, whereas GSTP1 silencing significantly weakened this induction, indicating that GSTP1 contributes to the oxidative stress signaling cascade activated by BioA. Similar to the results observed for SLC7A11 and HMOX1, NAC treatment reduced NQO1 mRNA expression across all groups, confirming that the transcriptional activation of antioxidant genes in response to BioA is largely mediated by ROS accumulation.

Taken together, these results demonstrate that BioA induces a robust oxidative stress response in glioma cells, characterized by the transcriptional activation of multiple antioxidant defense genes. GSTP1 depletion significantly attenuates this response, whereas ROS scavenging by NAC further suppresses antioxidant gene expression, supporting the notion that GSTP1 contributes to BioA-induced redox signaling and oxidative stress adaptation in glioma cells.

### Independent prognostic analysis and immune-related analysis

**Figure 10:**
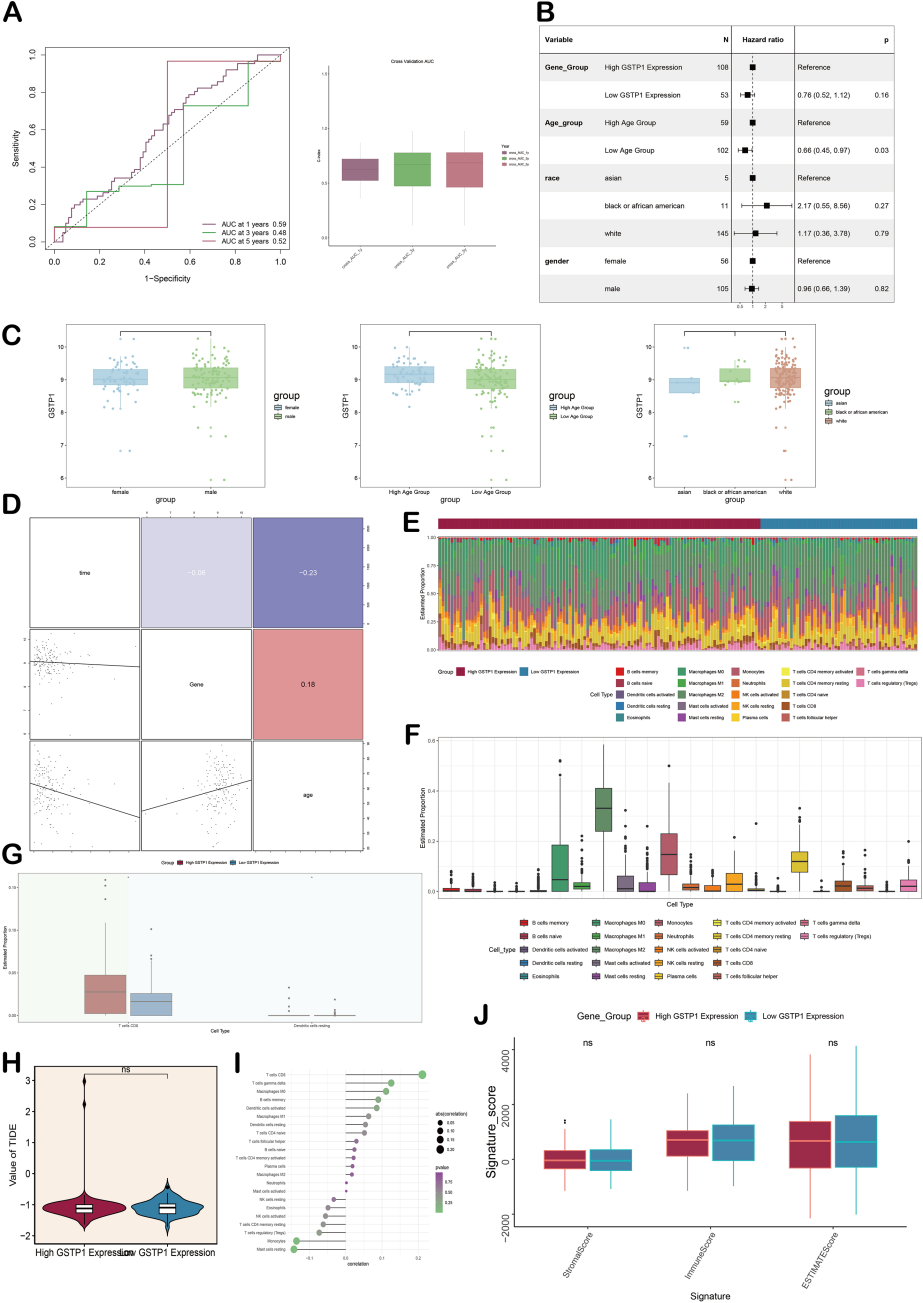
Independent prognostic analysis and immune infiltration analysis. **(A)** The above ROC curve was used to evaluate the accuracy of the patient’s survival prediction, showing the AUC values of 1 year, 3 years, and 5 years. The box plot below visualizes the C-index for cross-validation in 1, 3, and 5 years. **(B)** The forest plot showed the results of multivariate COX regression analysis, including gene group, age group, race, and gender. **(C)** The box plot showed *GSTP1* scores for different genders, age groups, and races. **(D)** The correlation matrix diagram revealed the relationship between time, gene, and age variables. **(E, F)** The stacking bar chart and box plot showed the estimated proportions of 22 immune cells in different *GSTP* score groups. **(G)** The box plot showed the estimated proportions of T cell CD8 and dendritic cells resting in the high and low *GSTP1* expression groups. **(H)** The violin plot showed the value of TIDE of high and low *GSTP1* expression groups. **(I)** The lollipop chart showed the direct correlation between different states of immune cells and risk. **(J)** Box plot analysis was used to compare the difference of stromal score, immune score, and ESTIMATE score between high and low *GSTP1* expression groups.

To further elucidate the prognostic implications for patients in the *GSTP1*-associated risk subgroup, we carried out a detailed and targeted clinical outcome analysis. The ROC curve and box plot evaluated the predictive ability of *GSTP1* for prognosis, and the results confirmed that it had a high predictive accuracy for survival rates (**Figure 10A**). The results of Cox regression revealed that being in the younger age group conferred a favorable prognosis for glioma patients, while race and sex had no significant impact on patient survival (**Figure 10B**). The box plot analysis demonstrated that the expression level of *GSTP1* was relatively lower in the low age group (**Figure 10C**). The correlation matrix diagram revealed the relationship between variables, in which ‘survival time’ was weakly negatively correlated with ‘*GSTP1* expression,’ and ‘*GSTP1* expression’ was weakly positively correlated with ‘age’ (**Figure 10D**).

For investigating the association between GSTP1 expression and the tumor immune microenvironment, the predicted immune cell proportions in the two *GSTP1* groups were presented via a stacked bar chart (**Figure 10E**). The important immune cells in two *GSTP1* expression groups included macrophages M0, etc. Then, CD8+ T cells were higher in the *GSTP1* high expression group (**Figures 10F, G**). Subsequent analysis showed a significantly higher TIDE score in the *GSTP1* high-expression group, suggesting that the patients’ tumor microenvironment was immunosuppressive, which may be an important cause of unfavorable prognosis (**Figure 10H**).

The relationship between the risk score and immune infiltration showed that higher scores were closely linked to CD8^+^ T-cell presence, whereas resting mast cells displayed an opposing, negative correlation. These results offer key evidence for investigating GBM progression and immune escape mechanisms (**Figure 10I**). The box plot demonstrated that the matrix score of the *GSTP1* high-expression group was higher, indicating stronger tumor heterogeneity in patients of this group (**Figure 10J**).

### Enrichment analysis and drug sensitivity analysis in bulk RNA-seq

**Figure 11:**
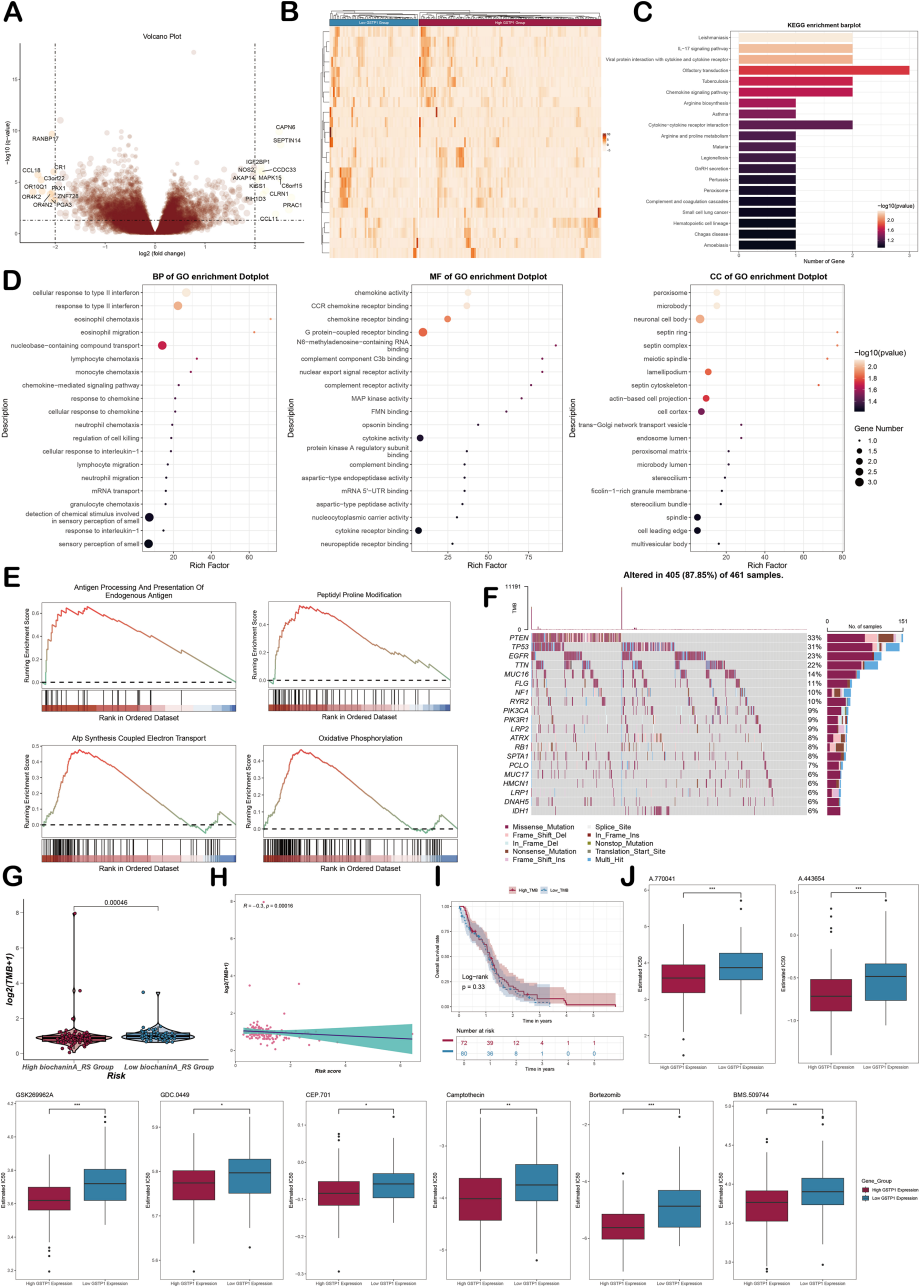
Enrichment analysis and drug sensitivity of the high *GSTP1* group and the low *GSTP1* group. **(A)** Volcano plots showed significantly up-regulated and down-regulated genes in the high *GSTP1* group and the low *GSTP1* group. **(B)** The heatmap showed the expression of DEGs in the high *GSTP1* group and the low *GSTP1* group. **(C)** The bar chart showed the enrichment of all KEGG functional pathways. **(D)** The bar chart showed the enrichment of GOBP, GOMF, and GOCC functional pathways. **(E)** GSEA plots showed functional pathways related to glioma. **(F)** The waterfall diagram showed the distribution of mutant genes and the difference in mutation frequency between the High *GSTP1* group and the Low *GSTP1* group. **(G)** The violin plot showed the expression of TMB in the high *GSTP1* group and the low *GSTP1* group. **(H)** The scatter plot showed the relationship between TMB and *GSTP1* risk score. **(I)** The survival curve showed the overall survival rate of high and low TMB. Red represents the high TMB group, and blue represents the low TMB group. **(J)** The box plot showed the difference in IC50 values of xx drugs between the high *GSTP1* group and the low *GSTP1* group.

In order to further explore the GBM from the bulk level, this study analyzed the DEGs in two *GSTP1* groups by bioinformatics methods such as GO, KEGG, and GSEA. The volcano plot intuitively showed the distribution characteristics of DEGs between two *GSTP1* groups. The results showed that *CAPN6, SEPTIN14*, and other genes were up-regulated in the *GSTP1* high expression group (**Figure 11A**). DEGs across the two groups were visualized in a heatmap to display overall expression trends (**Figure 11B**). KEGG enrichment analysis results demonstrated that DEGs were chiefly enriched in pathways such as Leishmaniasis. These signaling pathways were likely to have important regulatory functions in GBM progression (**Figure 11C**). GO enrichment analysis revealed enrichment of the cellular response to type II interferon, chemokines, peroxisomes, and other items (**Figure 11D**). GSEA revealed that DEGs were notably up-regulated in ATP synthesis coupled electron transport, endogenous antigen processing and presentation, peptidyl proline modification, oxidative phosphorylation, and other related pathways (**Figure 11E**). The waterfall diagram clearly illustrated the distribution of key mutated genes and the variation in mutation frequency across the two groups (**Figure 11F**). The low *GSTP1* group showed higher TMB values (**Figure 11G**). Studies have shown that TMB value is negatively correlated with *GSTP1*. Surprisingly, high TMB was associated with better prognosis (P = 0.33) (**Figures 11H, I**). The sensitivity of GBM-related drugs was analyzed by estimated IC50. Eight drugs (A.770041, A.443654, GSK269962A, GDC.0449, CEP.701, camptothecin, bortezomib, and BMS.509744) showed higher sensitivity in the *GSTP1* high-expression group. They were potential treatments for GBM patients with high *GSTP1* expression (**Figure 11J**).

## Discussion

Glioma, as a solid brain tumor, has attracted much attention due to its significant heterogeneity, strong invasiveness, treatment resistance, and poor prognosis. Its significant heterogeneity restricts the effectiveness of diverse therapeutic strategies, leading to drug resistance and subsequent recurrence. Within gliomas, the tumor microenvironment promotes both therapeutic resistance and the development of immune escape mechanisms. These factors collectively contribute to the poor prognosis observed in glioma patients. At present, understanding and overcoming glioma heterogeneity, identifying novel therapeutic targets, and modulating the immune microenvironment have become research priorities. Previous studies have shown that active Chinese medicine component bioA exerts antitumor effects (62–64). However, its potential target genes and mechanisms of action remain unclear, limiting its further application. In brief, multiple transcriptome sequencing analyses uncovered the molecular mechanisms of bioA in glioma.

ScRNA-seq identified eight glioma cell subpopulations. Among them, the C2 subgroup exhibited strong proliferative characteristics and was predominantly enriched in rGBM. The C2 subgroup may be of great significance for understanding glioma recurrence and identifying potential therapeutic targets. C2 subgroup showed marked cancer relevance, with key genes including *HIST1H4C* (65), *HMGB2* (66), *CENPF* (67), *UBE2C* (68), and *PTTG1* (69). Functionally, C2 was highly active in many important pathways (70–72). Further enrichment analysis displayed that the C2 subgroup was mainly enriched in mitosis, cell cycle, and transition, highlighting its high heterogeneity and proliferation potential.

During the differentiation process, the C2 subgroup also played a central role. It exhibited the highest stemness prediction score and the greatest differentiation potential, positioning it at the initiation site of the Monocle trajectory. Slingshot analysis further confirmed that other subpopulations originated from C2 subgroup, suggesting that it may act as a pivotal cluster driving glioma differentiation. Together, pseudotime analyses outlined the dynamic differentiation landscape of glioma, offering new insights into its developmental trajectory.

Cell–cell communication analysis revealed extensive crosstalk in many cell types. Notably, the C2 subgroup demonstrated strong interactions with ECs through the PTN signaling pathway. PTN, a heparin-binding growth factor, promotes tumor proliferation and angiogenesis (73) and has been demonstrated to be involved in the pathological progression of diverse types of malignancies. In breast cancer, PTN activated NF-κB signaling in cancer cells, modulates cytokine expression, suppresses the immune microenvironment, and drives metastasis (74). In prostate cancer, elevated PTN levels serve as markers of recurrence and metastasis (75). Moreover, the PTN pathway played a key role in glioma malignancy. We found that the PTN–NCL ligand–receptor pair was highly expressed in this pathway, consistent with reports showing that PTN–NCL interactions promote angiogenic activity (73), further confirming its functional significance.

Through the analysis of TFs, we investigated the core regulatory mechanisms of glioma. The top five TFs in C2 subgroup were all closely related to glioma progression. FoxM1 interacts with β-catenin to enhance β-catenin transcriptional activity and Wnt target gene expression, thereby promoting glioma malignancy (76). Overexpression of E2F1 markedly upregulates CENPM, induces angiogenesis and chromosomal instability or aneuploidy, and drives tumor growth and invasion (77). TFDP1 participates in cell cycle regulation and is closely associated with glioma progression (78). HDAC2 reshapes chromatin accessibility to sustain glioma growth and self-renewal (79). XBP1 regulates glycolysis in glioma cells; its silencing reduces viability and lactate production under hypoxic conditions, thereby inhibiting tumor formation (80).

To systematically elucidate the pharmacological mechanisms of bioA in glioma treatment, network pharmacology analysis was performed to predict and screen potential target genes. Topological and functional scoring analysis revealed the significant enrichment of core target genes in the C2 subgroup, supporting their core status in glioma. Further analysis identified *GSTP1* as a highly specific gene in the C2 subgroup, which was closely related to poor prognosis. Pathway enrichment showed that bioA target genes were enriched in oxidative stress–related pathways, including “response to reactive oxygen species” and “cellular response to oxidative stress,” aligning with the functional properties of *GSTP1*. Previous studies have shown that *GSTP1*, an oxidative stress regulatory gene, catalyzes the conjugation of glutathione to electrophilic compounds to maintain redox homeostasis; its loss exacerbates oxidative stress, activates MAPK and NF-κB pathways, and induces apoptosis (81, 82). These findings suggest that bioA may interfere with the *GSTP1*-mediated oxidative stress adaptation mechanism, thereby disrupting the metabolic advantage and proliferative balance of the C2 subgroup.

Additionally, *GSTP1* mediates oxidative stress and promotes GBM cell proliferation (83). Sesquiterpene lactones have also been reported to inhibit glioma progression by targeting *GSTP1 (*84). Sesquiterpene lactone derivatives from the traditional Chinese herb Elephantopus scaber have demonstrated promising anti-glioma potential in research. These active components are capable of inducing cell cycle arrest and inhibiting tumor angiogenesis (85). In order to further verify the role of GSTP1 in intracranial tumors, this study carried out a series of functional experiments *in vitro*. The results showed that GSTP1 knockout could significantly inhibit the proliferation and migration of glioma cells and promote apoptosis, confirming that GSTP1 could promote the malignant progression of glioma. This study also explored the regulatory relationship between GSTP1 and BioA in the oxidative stress pathway. It was found that BioA treatment could cause a large accumulation of ROS in glioma cells and reduce the ratio of GSH/GSSG, thereby triggering oxidative stress and inhibiting cell proliferation; GSTP1 knockout attenuated BioA-induced ROS production, partially restored GSH/GSSG ratio, alleviated cell redox imbalance, and significantly inhibited BioA-mediated transcriptional activation of oxidative stress-responsive genes such as SLC7A11, HMOX1, and NQO1. In addition, GSTP1 knockout combined with BioA treatment synergistically enhanced the inhibitory effect on glioma cell clone formation ability and cell viability, while the addition of NAC partially reversed the inhibitory effect.

The above results further confirmed that the C2 subgroup with high expression of GSTP1 could enhance its metabolic adaptability and proliferation potential through oxidative stress adaptation mechanism. In summary, GSTP1 is expected to be a potential target for the treatment of glioma. Targeted inhibition of GSTP1 may enhance the sensitivity of glioma cells to anti-tumor therapies such as BioA and other oxidative stress, and provide new ideas for the treatment of glioma. To clarify the prognostic implications for glioma patients, we constructed a *GSTP1*-based prognostic risk model. When integrated with immune infiltration analysis, the predictive capacity of the model was further verified. The model effectively distinguished patients with different prognostic risks. Moreover, risk scores were strongly positively correlated with T cells C8 and T cells γδ, suggesting that *GSTP1* may regulate immune cell function and suppress the immune microenvironment. This implies that bioA may exert its effects through dual mechanisms involving “oxidative stress regulation” and “immune microenvironment remodeling.” Functional enrichment analysis showed significant activation of Oxidative Phosphorylation (86) and ATP Synthesis Coupled Electron Transport pathways (87), further supporting *GSTP1* as a potential target. Subsequently, patients with elevated *GSTP1* expression exhibited greater sensitivity to CEP.701, providing a theoretical basis for potential combination therapies involving bioA and expanding therapeutic options for clinical application.

However, the research has certain limitations. Above all, network pharmacology analysis relies on databases that may lack completeness and accuracy, affecting the reliability of results. Secondly, the single-cell sequencing dataset had limited sample size. Thirdly, the function of *GSTP1* unvalidated *in vivo*. Future research should build upon this foundation and further employ animal models and molecular docking techniques for verification. The aim is to further transform biological A into clinical applications and lay a solid foundation for the development of treatment methods targeting *GSTP1*. To improve the prognosis of glioma patients, overcome the TME and the invasiveness of the tumor itself, new approaches will be provided.

## Conclusion

In the research, the core mechanism of active Chinese medicine component biological A in the treatment of brain tumors was systematically elucidated through multi-omics analysis. Firstly, through scRNA-seq, the key C2 subgroup with high proliferative activity and stemness was identified. This subpopulation regulates its malignant phenotype through the PTN signaling pathway, core TFs FOXM1 and E2F1. The bioA-related gene pathways were highly expressed in the C2 subgroup, with *GSTP1* being the core. The experimental results displayed that knocking out the *GSTP1* gene can achieve the result of inhibiting tumors. At the same time, it was found that GSTP1 is a key target of biological A regulating oxidative stress pathway, and the combination of the two can synergistically enhance the anti-tumor effect. Finally, to understand the specific conditions of patients with different *GSTP1* expression groups, we created a relevant prognostic model, providing personalized treatment options for clinical treatment of glioma. In conclusion, *GSTP1* was a key target for biological A in the treatment of brain tumors, providing a theoretical basis and new strategies for precise immunotherapy of brain tumors.

## Data Availability

The original contributions presented in the study are included in the article/**Supplementary Material**. Further inquiries can be directed to the corresponding authors.

## Supplementary material

Supplementary Figure 1 **(A)**. The Ro/e score was used to evaluate the tissue preference of five cell types for different phases and groups. **(B)**. Volcano maps showed significantly up-regulated and down-regulated genes in five cell types. **(C)**. The word cloud map showed the key functions in the five cell types. The larger the font, the greater the count. Red represents a high score, and blue represents a low score. **(D)**. GSEA maps showed the up-regulated enrichment pathway of glioma.

Supplementary Figure 2 InferCNV analysis, with ECs as reference cells, the copy number variation of glioma.

Supplementary Figure 3 **(A)**. The UMAP diagrams visualized each glioma cell subgroup and group (based on the activity score of the regulatory module). **(B)**. Scatter plots showed the specificity score of the top TFs in each glioma cell subgroup.

Supplementary Figure 4 The ClueGO software revealed the biological pathways that are significantly associated with the bioA.
