# Supplementary figures and images for: The therapeutic effect and specific mechanism involved active Chinese medicine component biochaninA in glioma
Source: Front Immunol. 2026 Apr 21;17:1780159. doi: 10.3389/fimmu.2026.1780159 (PMC13139340; doi:10.3389/fimmu.2026.1780159)

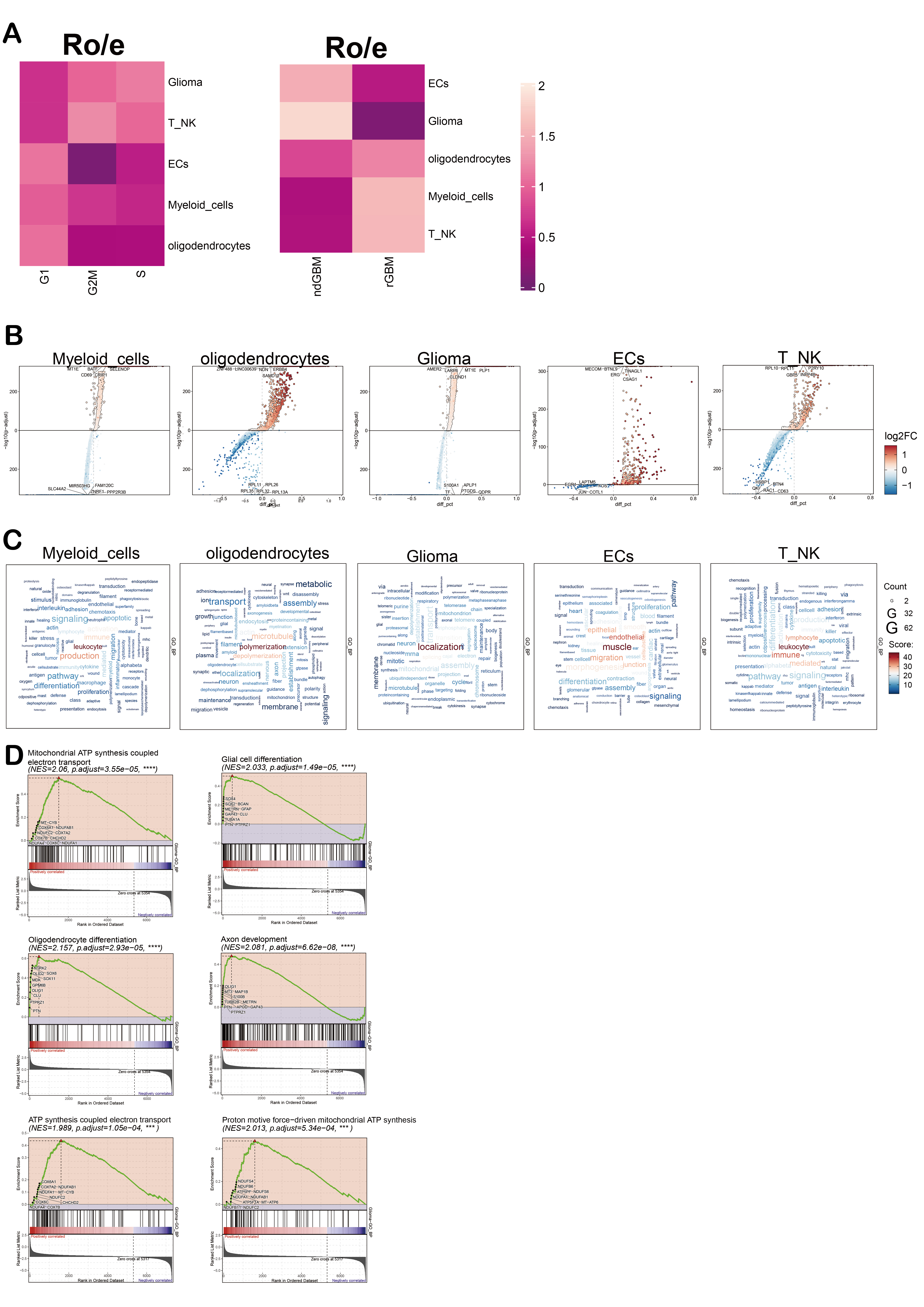

Supplement: Supplementary Figure 1 — (A). The Ro/e score was used to evaluate the tissue preference of five cell types for different phases and groups. (B). Volcano maps showed significantly up-regulated and down-regulated genes in five cell types. (C). The word cloud map showed the key functions in the five cell types. The larger the font, the greater the count. Red represents a high score, and blue represents a low score. (D). GSEA maps showed the up-regulated enrichment pathway of glioma. [file Image1.jpeg]

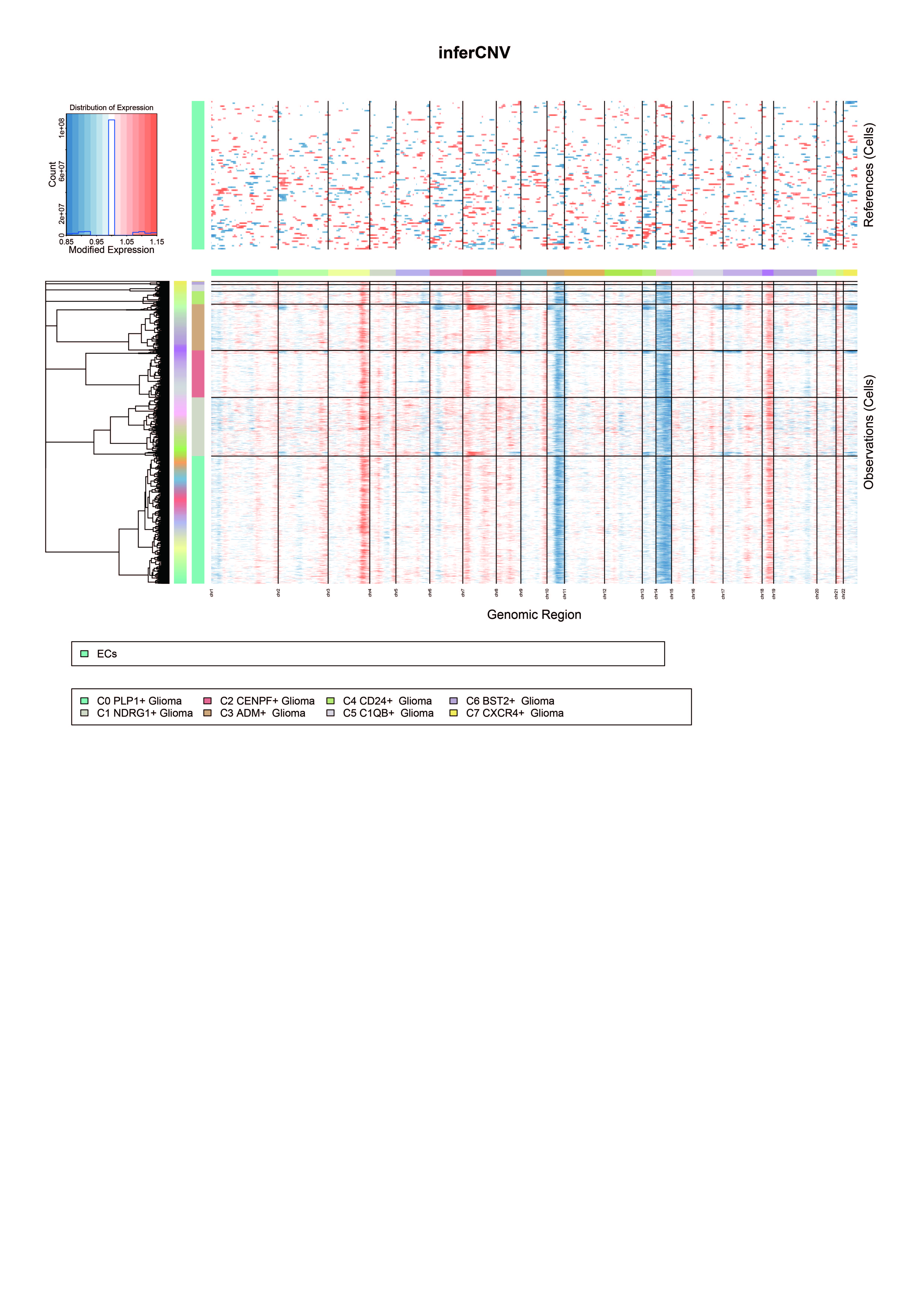

Supplement: Supplementary Figure 2 — InferCNV analysis, with ECs as reference cells, the copy number variation of glioma. [file Image2.jpeg]

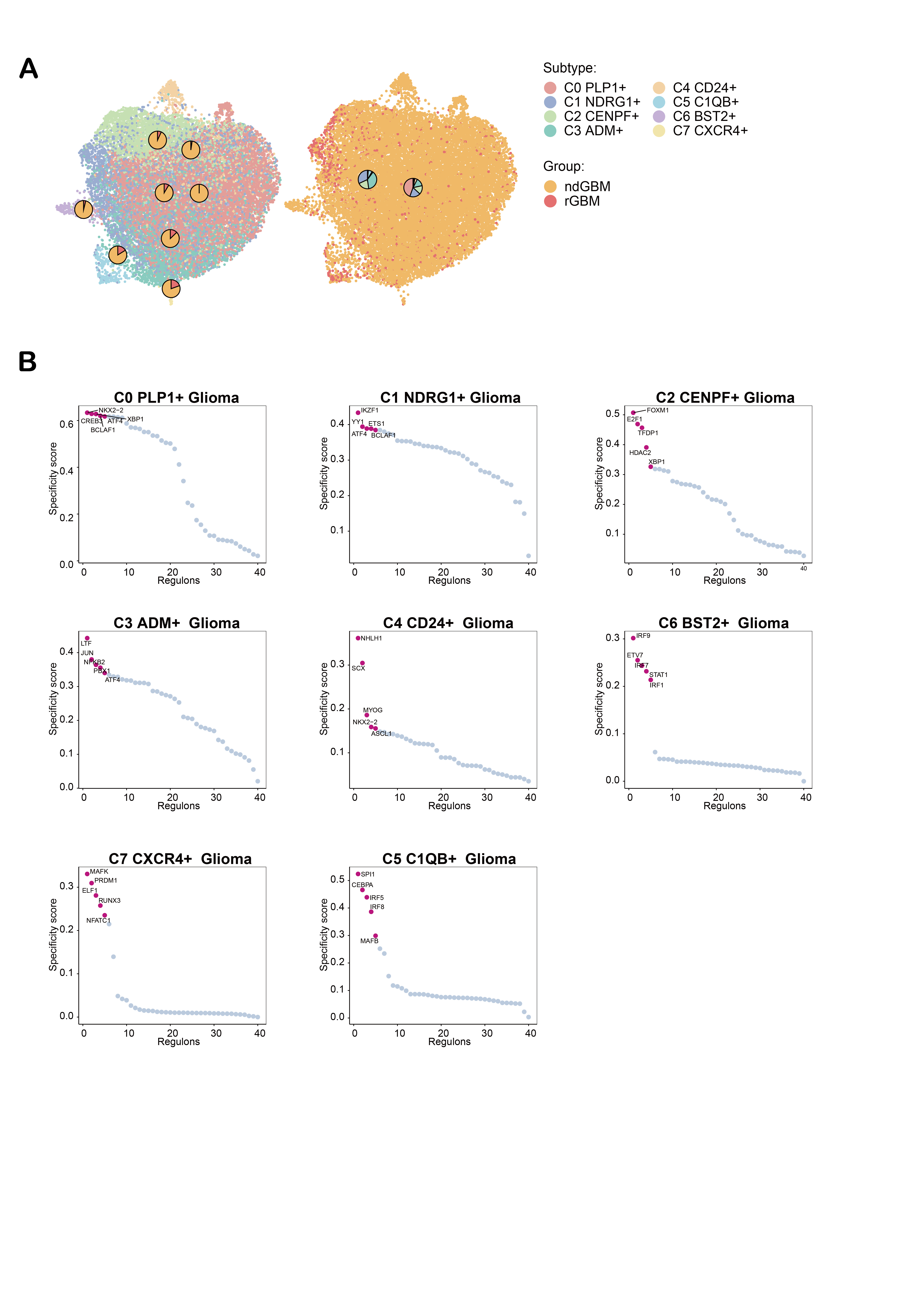

Supplement: Supplementary Figure 3 — (A). The UMAP diagrams visualized each glioma cell subgroup and group (based on the activity score of the regulatory module). (B). Scatter plots showed the specificity score of the top TFs in each glioma cell subgroup. [file Image3.jpeg]

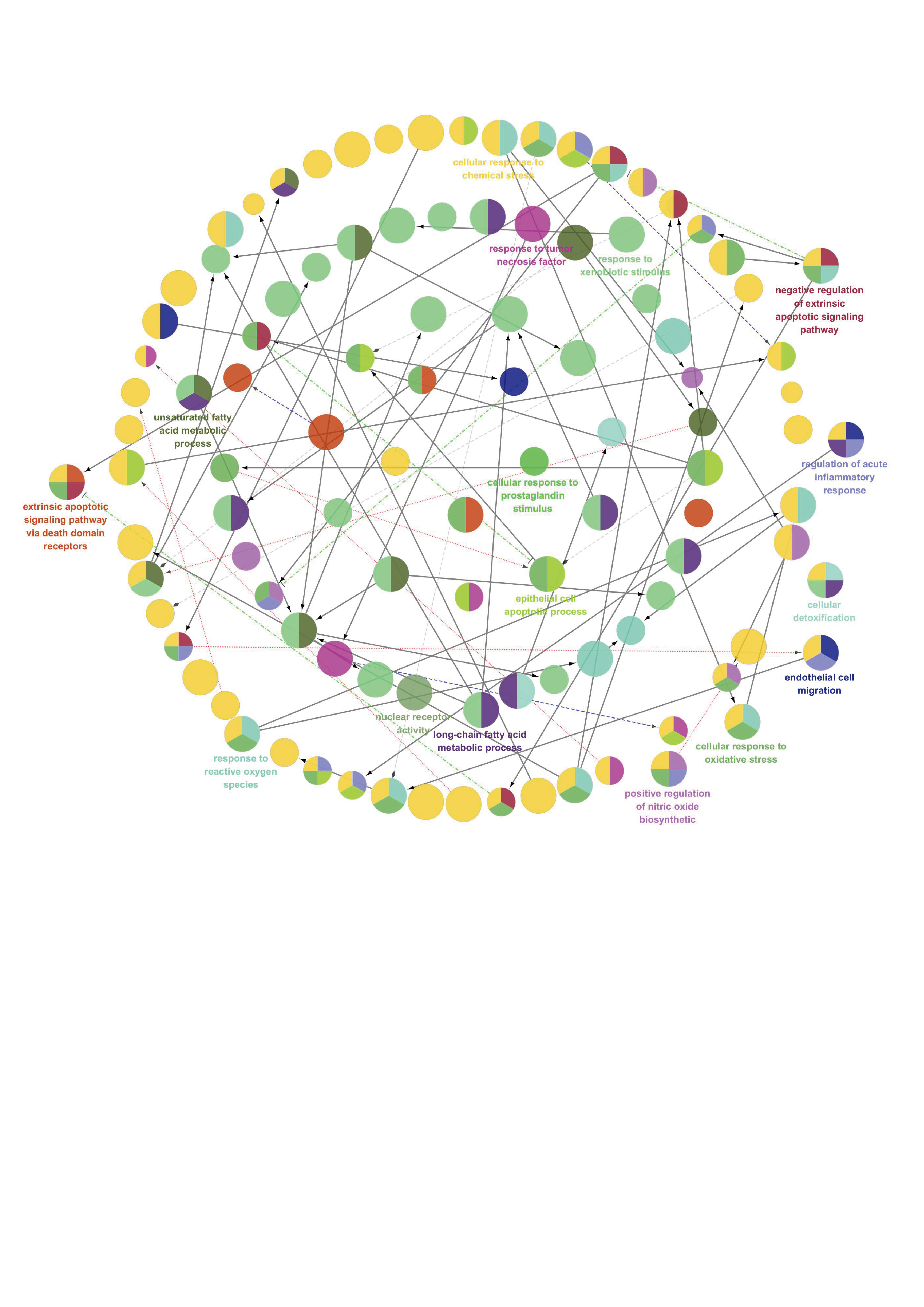

Supplement: Supplementary Figure 4 — The ClueGO software revealed the biological pathways that are significantly associated with the bioA. [file Image4.jpeg]
